# α-Amino-3-hydroxyl-5-methyl-4-isoxazole-propionate receptor and RNA processing gene dysregulation are early determinants of selective motor neuron vulnerability in a mouse model of amyotrophic lateral sclerosis

**DOI:** 10.1093/braincomms/fcac081

**Published:** 2022-03-31

**Authors:** Pardis F. Zanganeh, Samantha K. Barton, Katherine Lim, Elizabeth L. Qian, Duncan E. Crombie, Christopher R. Bye, Bradley J. Turner

**Affiliations:** 1 The Florey Institute of Neuroscience and Mental Health, University of Melbourne, Melbourne, VIC 3052, Australia; 2 The Perron Institute for Neurological and Translational Science, Queen Elizabeth Medical Centre, Nedlands, WA 6150, Australia

**Keywords:** AMPA receptor, amyotrophic lateral sclerosis, motor neurons, transcriptomics, RNA processing

## Abstract

Amyotrophic lateral sclerosis is a late-onset adult neurodegenerative disease, although there is mounting electrophysiological and pathological evidence from patients and animal models for a protracted preclinical period of motor neuron susceptibility and dysfunction, long before clinical diagnosis. The key molecular mechanisms linked to motor neuron vulnerability in amyotrophic lateral sclerosis have been extensively studied using transcriptional profiling in motor neurons isolated from adult mutant superoxide dismutase 1 mice. However, neonatal and embryonic motor neurons from mutant superoxide dismutase 1 mice show abnormal morphology and hyperexcitability, suggesting preceding transcriptional dysregulation. Here, we used RNA sequencing on motor neurons isolated from embryonic superoxide dismutase 1^G93A^ mice to determine the earliest molecular mechanisms conferring neuronal susceptibility and dysfunction known in a mouse model of amyotrophic lateral sclerosis. Transgenic superoxide dismutase 1^G93A^ mice expressing the spinal motor neuron homeobox HB9:green fluorescent protein reporter allowed unambiguous identification and isolation of motor neurons using fluorescence-activated cell sorting. Gene expression profiling of isolated motor neurons revealed transcriptional dysregulation in superoxide dismutase 1^G93A^ mice as early as embryonic Day 12.5 with the majority of differentially expressed genes involved in RNA processing and α-amino-3-hydroxyl-5-methyl-4-isoxazole-propionate-mediated glutamate receptor signalling. We confirmed dysregulation of the α-amino-3-hydroxyl-5-methyl-4-isoxazole-propionate receptor Subunit 2, at transcript and protein levels, in embryonic superoxide dismutase 1^G93A^ mouse motor neurons and human motor neurons derived from amyotrophic lateral sclerosis patient induced pluripotent stem cells harbouring pathogenic superoxide dismutase 1 mutations. Mutant superoxide dismutase 1 induced pluripotent stem cell-derived motor neurons showed greater vulnerability to α-amino-3-hydroxyl-5-methyl-4-isoxazole-propionate-mediated excitotoxicity, consistent with α-amino-3-hydroxyl-5-methyl-4-isoxazole-propionate receptor Subunit 2 downregulation. Thus, α-amino-3-hydroxyl-5-methyl-4-isoxazole-propionate receptor Subunit 2 deficiency leading to enhanced α-amino-3-hydroxyl-5-methyl-4-isoxazole-propionate receptor signalling, calcium influx, hyperexcitability, and chronic excitotoxicity is a very early and intrinsic property of spinal motor neurons that may trigger amyotrophic lateral sclerosis pathogenesis later in life. This study reinforces the concept of therapeutic targeting of hyperexcitability and excitotoxicity as potential disease-modifying approaches for amyotrophic lateral sclerosis.

## Teaser

Zanganeh *et al*. report the earliest known transcriptional dysregulation in motor neurons from embryonic mutant superoxide dismutase 1 mice and amyotrophic lateral sclerosis patients. They conclude that glutamate receptor excitotoxicity and RNA processing defects are early determinants of cell vulnerability in amyotrophic lateral sclerosis.

## Graphical Abstract

**Graphical Abstract:**
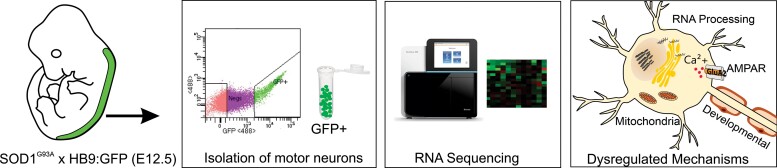

## Introduction

Amyotrophic lateral sclerosis (ALS) is an adult-onset neurodegenerative disease that is caused by the loss of both upper and lower motor neurons. ALS results in rapidly progressive physical disability, leading to fatal paralysis for which there is no effective treatment or cure.^1,2^ The majority of ALS cases are sporadic (SALS), whereas the remaining 10% are inherited (familial ALS or FALS). Dominant mutations in the superoxide dismutase 1 (SOD1) gene account for 20% of FALS and transgenic mice expressing mutant SOD1 remain the most widely used, best understood and accepted mouse model of ALS.^3^

Although ALS typically presents in mid-to-late life, there are multiple lines of evidence suggesting the existence of a protracted preclinical period before symptom onset in ALS patients and mouse models. First in patients, asymptomatic SOD1 mutation carriers exhibited both a reduction in motor unit number estimation using electromyography, indicative of preclinical motor neuron loss.^4^ Increased cortical hyperexcitability detected by threshold tracking transcranial magnetic stimulation which reflects upper motor neuron dysfunction also occurs in presymptomatic SOD1 mutation carriers which progress to ALS.^5^ In SOD1^G93A^ mice that develop symptom onset at postnatal Day 90 (P90), it is well established that peripheral synapses of spinal motor neurons degenerate as early as P45^6^ which was subsequently predated to P25,^7^ preceding distal axon loss at P80 and cell body loss at P120.^6^ At a subcellular level, Golgi apparatus fragmentation at P30^8^ and mitochondrial swelling at P14^9^ are early features of spinal motor neuron pathology in SOD1^G93A^ mice. At a synaptic level, dendritic pathology and increased synaptic neurotransmission in spinal motor neurons occur at ∼P8 in SOD1^G93A^ mice;^10^ however, the molecular basis of this hyperexcitability remains unclear. Finally, at a molecular level, mTOR activation at P15, mutant SOD1 aggregation at P7^11^ and endoplasmic reticulum (ER) stress at P5^12^ are the earliest reported dysfunctions in spinal motor neurons in SOD1^G93A^ mice.

Collectively, these findings in ALS patients and mouse models provide evidence of cell vulnerability and dysfunction before symptom onset in motor neurons and suggest the existence of a long-lasting disease process that may commence years, or potentially even decades, before clinical deficits. In support of this, Eisen *et al*.^13^ recently proposed that ALS onset may even date back to the perinatal period of life and conception, based on the embryonic expression of ubiquitous ALS-linked genes. These authors suggested that a combination of genetic, epigenetic and exogenous factors, coupled with metabolic and oxidative stress associated with early motor neuron development, may render motor neurons inherently vulnerable, thereby increasing the risk of ALS later in life. Indeed, there is evidence for abnormalities in spinal motor neurons cultured from embryonic SOD1^G93A^ mice shown by enhanced hyperexcitability^14^ and axonal transport deficits,^15^ although these are cell culture experiments and evidence is lacking *in vivo*.

We, therefore, sought to test this provocative hypothesis using an unbiased transcriptomic approach in spinal motor neurons of embryonic SOD1^G93A^ mice. While gene expression profiling of isolated motor neurons has been extensively performed in mutant SOD1 mice uncovering potential candidate disease pathways,^12,16–18^ these studies have been restricted to adult (P30–P120) or early postnatal (P5) mice using laser-capture microdissection and microarray analysis. Here, we devised a novel methodology to isolate enriched populations of spinal motor neurons from embryonic SOD1^G93A^ mice. We coupled fluorescence-activated cell sorting (FACS)-based isolation and purification of motor neurons from SOD1^G93A^ mice co-expressing a well-validated fluorescent motor neuron reporter, homeobox HB9 (HB9):green fluorescent protein (GFP),^19^ with highly sensitive transcriptional profiling to identify early changes in gene expression in embryonic wild-type (WT) and SOD1^G93A^ mice. A distinct gene expression profile was identified as early as embryonic Day (E) 12.5 in SOD1^G93A^ mice. We identified significant changes in genes associated with excitotoxicity and RNA processing and validated the involvement of these molecular mechanisms at transcriptional and protein levels. Importantly, these findings were confirmed in induced pluripotent stem cell (iPSC)-derived spinal motor neurons from SOD1-linked ALS patients carrying three distinct mutations which accordingly showed greater vulnerability to excitotoxicity. Our study identifies dysregulation of α-amino-3-hydroxyl-5-methyl-4-isoxazole-propionate (AMPA) receptor signalling and RNA processing genes as very early, intrinsic and key contributors linked to motor neuron susceptibility in ALS *in utero*.

## Materials and methods

### Animals

All animal procedures were performed in accordance with the guidelines of the Australian National Health and Medical Research Council’s published Code of Practice for the use of animals in research and were approved by the Florey Institute of Neuroscience and Mental Health Animal Ethics Committee (permit number: 16-026). Transgenic HB9:GFP mice [B6.Cg-Tg(Hlxb9-GFP)1Tmj/J, stock no. 005029]^19^ and SOD1^G93A^ mice [B6.Cg-Tg(SOD1*G93A)1Gur/J line; stock no. 004435; RRID:IMSR_JAX:004435] were obtained from the Jackson Laboratory (Bar Harbor, ME, USA) and maintained on C57BL/6J backgrounds. SOD1^G93A^ male and HB9:GFP female mice were crossed to generate SOD1^G93A^; HB9:GFP^+/−^ and HB9:GFP^+/−^ mice, which were subsequently crossed to generate experimental animals: SOD1^G93A^; HB9:GFP^+/+^ and HB9:GFP^+/+^ mice. HB9:GFP^+/+^ mice were henceforth called WT for this study. Mice were maintained under standard conditions of 12 h light/dark cycles with access to food and water *ad libitum*. Mice were time-mated overnight, and embryonic (E) Day 0.5 was designated following visualization of a vaginal plug the next morning.

### DNA extraction and genotyping

Tissue for DNA extraction was obtained from embryo tail. DNA was extracted using a rapid genotyping method using red extract-N-AmpTM Tissue PCR Kit (Sigma–Aldrich; 254-457-8). Two μl of diluted DNA was added to the master mix containing 100 nM forward and reverse primers, nuclease-free water and 5 μl of SsoAdvanced universal SYBR Green Supermix (BioRad; 1725274). Samples were then genotyped on a real-time PCR machine (BioRad; CFX96), targeting human SOD1 using forward (5′-CATCAGCCCTAATCCATCTGA-3′) and reverse primers (5′-CGCGACTAACAATCAAAGTGA-3′). The thermal cycling condition was set as the following: 2 min denaturation at 95°C, annealing at 95°C for 5 s and extension at 60°C for 5 s for 40 cycles.

### Isolation of spinal motor neurons

Pregnant mice were deeply anaesthetized with 3% isoflurane inhalation (Delvet, Seven Hills, NSW, Australia) and administered sodium pentobarbital (11 mg/kg 0.1 ml, i.p.). Lumbar spinal cords were microdissected from HB9:GFP and SOD1^G93A^ HB9:GFP embryos at E12.5 in chilled Leibovitz’s L-15 medium (Gibco™; 11415064). Littermate lumbar spinal cords of the same genotype were pooled (2–3 per genotype) and dissociated into a single-cell suspension using a neural tissue dissociation kit (Miltenyi Biotec; 130-094-802) according to the manufacturer’s instructions. Cell viability and density were determined by trypan blue exclusion counting. Cells were resuspended in 500 μl of HBSS^Ca2^^+^ ^Mg2^^+^ containing 1% (v/v) bovine serum albumin (Sigma–Aldrich; 9048-46-8) and the cell suspension filtered using a 40 μm cell strainer. Sorting was conducted on a FACS Aria^TM^ Fusion Flow Cytometer (Becton Dickinson) using 85 μm nozzle size, 45 psi sheath pressure, 1.0 flow rate and 488 nm optical path/laser. Dead cells and doublets were excluded using side scatter and forward scatter parameters based on DAPI staining and gating for the collection of GFP+ cells was set using GFP− tissue. Sorted GFP+ cells were directly collected in RLT lysis buffer (Qiagen; 74004) and the lysates were stored at −80°C.

### RNA extraction and qRT-PCR

Total RNA was isolated from cell lysates using a RNeasy Micro kit (Qiagen; 74004), including an on-column DNase I (Qiagen) digestion step according to the manufacturer’s instructions. RNA quantity and integrity were assessed by a 5200 Fragment Analyzer system (Agilent Technologies Ltd). First-strand cDNA was synthesized from 50 ng of total RNA using a reverse transcription reagent kit according to the manufacturer (PrimeScriptTM RT; Takara; RR037A). Primers for each candidate gene were designed using Primer3^20^ and are shown in Table 1. Real-time qPCR was carried out using SYBR® Premix Ex TaqTM II (Takara; RR820L) and 10 μM primer with 4–5 biological replicates for each group. Relative gene expression normalized to mouse *Gapdh* was determined using the ΔΔCt method.^21^ Droplet digital PCR and restriction fragment length polymorphism (RFLP) analyses are described in Supplementary Tables 1 and 2.

**Table 1:** List of primers used in this study for quantitative RT-PCR

Gene	Forward primer (5′–3′)	Reverse primer (5′–3′)
***Adarb1***	AGTGAGGGTCTTCAGTTGCA	TGCATGAGGGGAGGAAAAGT
***Flrt2***	AAATGGGCCACAGTCTCGTA	CGGTAGTTGAACGCATCCAG
***Gfap***	TAAGCTAGCCCTGGACATCG	GCCTTCTGACACGGATTTGG
***Gria1***	TGCTTTGTCACAACTCACGG	ATGGCGTACACTCCTTTGGA
***Gria2***	AATACCCTGGAGCACACACA	CCTCTGCTTCCGAAGATTGC
***Gria3***	ACAAAGCCCTCCTGATCCTC	GTGAAGAACCACCAAACCCC
***Gria4***	TGTTGGGAAGCACGTCAAAG	TCGTCACCATGGGCGTATTA
***Mbp***	TACCCTGGCTAAAGCAGAGC	CAGGGAGCCATAATGGGTAG
***Mnx1***	GTTGGAGCTGGAACACCAGT	CTTTTTGCTGCGTTTCCATT
***Pemt***	TGCTTTTGAACATCCTCCGC	CTGGACAGCACAAACACGAA
***Slc17a7 (Vglut1)***	CTGGGGTCCTTGTGCAGTAT	GTATTTGCGCTCCTCCTCAG
***Gas5***	GGTTTAAGTGGACGCAGCAA	CCATCACAGAGGTCCACACT
***Isac2***	CTTTCCCAGGCTTTTGACCC	TGCAGCCTGAGGAATTCTGA
***Malat1***	CCAGTTTCCCCAGCTTTTCC	TACTGGCTGCATCAAGGTGA
***Meg3***	CGGAGGACACTTGGACTCTT	TCGATGGAGAAGAGCGAGTC
***Pnisr***	CATCTCCAGCTCCCCAAGAA	GCGCATCTTTAGCCACGTAA
***Ankr16***	GATGCAATTCAGTGTGGCCA	CTTCATCCTGCCCAGTGACT
***Mir124-1***	TGGGAGGGGTGCATTGTTAT	TGGGAAGGGGATCTGGTAGA
***ND4L***	GCTCCATACCAATCCCCATCA	TCTGTTCCGTACGTGTTTGA
***Cpne6***	GACTTCATCGGCGAGTTCAC	CTTGTCTCGGTACTTGGGGT
***Ankrd23***	CTGGTGAACAAGCTGCTGG	TTGTCTTGTGCGTTGACCTG
***Adamts10***	AGTGCCTGTGAGACCATTGA	GTGACTCAGGGACAGGTTCA
***Appl2***	CACGACCTGTCAATGGCAAA	CGTTGAGTGCGCAGTAGTAC
***Rmst***	ACTGAAGAGTACAACCGCCA	GCCTTATTGCTGCTCCTGTC
***Gapdh***	GGTGCTGAGTATGTCGTGGA	GTGGTTCACACCCATCACAA
***Human-GRIA2***	CAAACAGCTTCGCAGTCACT	TGAAGGAGACGTGGAGTGTT
***Human-ADAR2***	TGTACATCAGCACCTCTCCC	TTTTGGTCCGTAGCTGTCCT
***Human 18S***	GTAACCCGTTGAACCCCATT	CCATCCAATCGGTAGTAGCG
***Human GAS5***	GAGCAAGCCTAACTCAAGCC	CCCAAGCAAGTCATCCATGG
***Human ISCA2***	TCAGAATTCCTCAGGCTGCA	GCCCCACCCTGTTCAAATAC
***Human MALAT1***	GGCTCAGTTGCGTAATGGAA	GAATTCGATCACCTTCCGCC
***Human MEG3***	ATCATCCGTCCACCTCCTTG	CAGTGAGTGGCTGCTTTGTA
***Human PNSIR***	AGATGCTGAAGGAGGGGATG	AACTGGGGATGGACTTCTGG
***Human ANKR16***	CACATTGCCAGTCGAGAAGG	CCTTGACTGCCTCCAAATGG

### RNA-seq library preparation and transcriptome analysis

RNA-seq libraries were prepared using the NeuGen Ovation® SoLo kit (0407-32) and subjected to 150 bp, paired-end sequencing on an Illumina HiSeq 2000 sequencer with four independent biological replicates analysed per group (four litters/collections per genotype). Reads were aligned to a reference mouse genome (mm10) and human genome (hg38) for identification of the human SOD1 transgene, both obtained from the University of California Santa Cruz using HISAT2 (2.1.0), under default parameters, with the exception of reporting only unique reads mapping to a unique location on the reference genome.^22^ The relative abundance of transcripts was measured using the FeatureCounts function in the Subread package.^23^ Differential expression analysis and principal component analysis (PCA) were conducted with DeSeq2 in RStudio (3.6.0).^24^ Gene ontology and pathway analysis were performed using the Database for Annotation, Visualization and Integrated Discovery (DAVID) functional tool^25^ and Ingenuity Pathway Analysis (IPA; QIAGEN), respectively.

### Immunohistochemistry

Embryonic lumbar spinal cords were fixed in 4% (w/v) paraformaldehyde (PFA) in 0.1 M phosphate buffer (PB) and picric acid at pH 7.2 for 2 h. Spinal cords were transferred to a 20% (w/v) sucrose in 0.1 M PB overnight at 4°C. Samples were embedded in OCT compound (Pro-SciTech, Queensland, Australia) and snap-frozen in isopentane on dry ice. Spinal cords were cross-sectioned at a thickness of 20 μm using a cryostat and placed on glass slides in series of 1:6 for immunostaining. For immunohistochemistry, the sections were rinsed with phosphate buffer saline (PBS) and blocked with 10% (v/v) normal donkey serum diluted in PBS with 0.3% (v/v) Triton X-100 for 1 h. The sections were incubated overnight at room temperature with primary antibodies. Primary antibodies were as follows: chicken anti-GFP (1:1000; Abcam; AB13970), rabbit anti-NeuN (1:1000; Abcam; AB104225), goat anti-ChAT (1:500; Abcam; AB34419) and guinea pig anti-AMPA receptor 2 subunit (GluA2) (1:500; Alomone Labs; AGP-073). On the following day, the sections were incubated in a secondary antibody (1:250) for 2 h at room temperature. Secondary antibodies were as follows: donkey, anti-chicken Alexa Fluor® 488 (Jackson ImmunoResearch Labs; 703-545-155), donkey, anti-guinea pig Alexa Fluor® 594 F(ab’)^2^ (Jackson ImmunoResearch Labs; 706-586-148) and anti-rabbit Alexa Fluor® 647 (Jackson ImmunoResearch Labs; 711-605-152). The sections were cover slipped using fluorescence mounting medium (DAKO; S3023). The slides were left to dry overnight in the dark at room temperature before imaging.

### Microscopy

Images were captured using an LSM 780 Zeiss confocal laser scanning microscope under 40× magnification. Quantification of GluA2 signal intensity was determined in Zen Blue software (ZEN lite, Zeiss) by measuring signal intensity of HB9:GFP-positive motor neurons. Five sections of L4 lumbar spinal cord (equivalent to 10 ventral horns) were selected at equally spaced intervals per animal with four mice per genotype. To compare signal intensity between different samples, the images were taken at the same excitation light intensity, exposure time and gain settings.

### Generation of iPSC-derived motor neurons

Patient iPSC lines containing SOD1^G85S^, SOD1^L144F^ or SOD1^I114T^ mutations and control line 11b^26^ were either generated in-house or kindly donated by Dr Kevin Eggan (Harvard Stem Cell Institute, MA, USA) and differentiated into spinal motor neurons as previously described.^27^ At Day 28, motor neurons were defined as mature by the expression of ChAT and Tuj1. RNA was harvested from mature motor neurons using RNeasy Micro kit (Qiagen; 74004) or the cells were fixed for immunocytochemistry.

### Immunocytochemistry

Cells were fixed for 10 min in 4% (w/v) PFA and washed twice in DPBS (ThermoFisher Scientific). Cells were blocked in 10% (v/v) normal donkey serum with 0.1% (v/v) Triton-X 100 in 0.1 M PBS and incubated overnight at 4°C in the following primary antibodies: goat anti-ChAT (1:500, Millipore; AB144P), chicken anti-β III tubulin (1:1000, Abcam; AB41489) and rabbit anti-GluA2 (1:500; Alomone Labs; AGC-005). Cells were incubated in the following secondary antibodies in the same diluent (1:400), for 2 h at room temperature; anti-goat Alexa Fluor®-488 (Jackson ImmunoResearch Labs; 705-545-147), anti-rabbit Cy™-550 (Jackson ImmunoResearch Labs; 711-165-152), anti-chicken Alexa Fluor®-647 (Jackson ImmunoResearch Labs; 703-605-155); followed by 15 min incubation in 1 μg/ml Hoechst 33342 (LifeTech; H1399). Cells were imaged at 20× magnification on Zeiss fluorescence microscope (Carl Zeiss AG, Germany). GluA2 expression was quantified using Zen Blue software (ZEN lite, Zeiss) by manual tracing of the cell body of motor neurons, using a minimum of 50 cells per biological replicate (*n* = 3 biological replicates per line). The intensity values were then subtracted from the background signal. The background signal was calculated as the mean of the pixels outside of a cell. To compare signal intensity between different lines, the images were taken at the same excitation light intensity, exposure time and gain settings.

### Excitotoxicity assay

Cryopreserved Day 19 motor neurons were seeded on laminin-521-coated 96-well plates at 1 × 10^5^ cells/well and cultured for 24 h in BrainPhys™ medium supplemented with 0.5 mM retinoic acid, 0.1 mM purmorphamine, 0.1 mM compound E, 20 ng/ml IGF1, 20 ng/ml CNTF1, 20 ng/ml BDNF and 10 mM Y-27632. An assay was performed on Day 28 motor neurons. Live cells were labelled with 2 μM Calcein Red™ (AAT Bioquest; 21900) and 0.02% (w/v) Pluronic® F-127 according to the manufacturer’s instructions at 37°C for 30 min and imaged using a Zeiss Axio fluorescence microscope (Zeiss AG, Germany) under 10× magnification. Cells were then treated with 100 μM of AMPA (Tocris Bioscience; 0254) for 24 h. Following treatment, cells were labelled again with Calcein Red™ and imaged. Cells were counted after treatment using Zen Blue image analysis software (ZEN lite, Zeiss) by counting two fields of view per well.

### Statistical analyses

All quantitative data are represented as means ± standard error of the means (SEMs). Statistical significance testing was performed using an unpaired Student’s *t*-test for comparison of two groups or one-way ANOVA with Dunnett’s multiple comparisons test for three or greater groups using GraphPad Prism 7.0 (GraphPad, USA).

### Data availability

The RNA-seq data generated from this study have been deposited in GEO accession number GSE142654.

## Results

### Transcriptional profiling of spinal motor neurons of embryonic Day 12.5 SOD1^G93A^ mice

**Figure 1:**
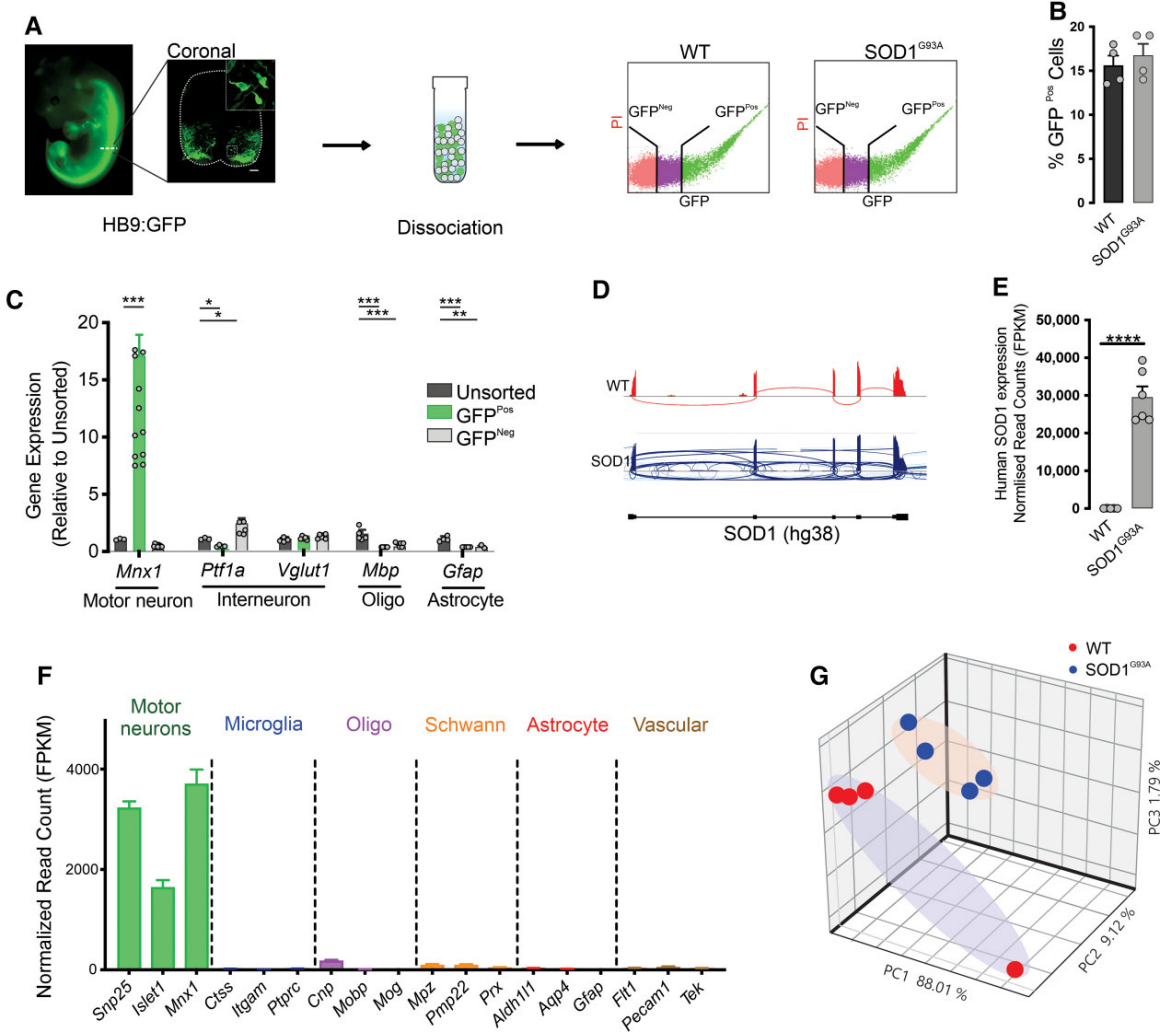
**Gene expression profiling of spinal motor neurons of embryonic SOD1^G93A^ mice**. (**A**) Representative images of HB9:GFP-SOD1^G93A^ transgenic mice at E12.5 and immunolabelling of HB9:GFP in a transverse section of spinal cord. Scale bar, 50 μm. Lumbar spinal cords were dissociated into single-cell suspension and motor neurons were isolated using FACS. (**B**) Bar graph representing the percentage of GFP+ cells in SOD1^G93A^ and WT samples. *n* = 4 biological replicates. (**C**) RNA was extracted from the sorted motor neurons, and purity of samples was examined using qRT-PCR by testing the expression for motor neuron, interneuron, oligodendrocyte and astrocyte gene markers. *n* = 4–11 biological replicates. (**D**) RNA sequencing reads were aligned to the human genome to evaluate the expression level of human SOD1 in samples. (**E**) Quantification of number of reads mapped to human SOD1. *n* = 6 biological replicates. (**F**) Assessment of sample purity using RNA sequencing normalized read counts for the expression of neuronal and glial marker genes. *n* = 12 biological replicates. (**G**) Clustering of gene expression profiles of spinal motor neurons from WT and SOD1^G93A^ mice were compared by PCA. Data represent mean ± SEM, unpaired student *t*-test, *n* = 4 biological replicates. *P < 0.01, **P < 0.001, ***P < 0.0001, ****P < 0.00001; GFP^Neg^, GFP-negative; GFP^Pos^, GFP-positive.

To date, all gene expression profiling studies of motor neurons in ALS have been conducted in post-mortem clinical endstage patient samples or postnatal often adult mouse models. To identify the earliest molecular pathways in SOD1^G93A^ mice that may underlie cell vulnerability to ALS, the transcriptional profile of lumbar spinal cord motor neurons was evaluated at E12.5. E12.5 represents motor neuron birth in the spinal cord, when motor neuron progenitors have exited the cell cycle and moved to their final position along motor pools.^28,29^ To specifically profile motor neurons in the spinal cord, HB9:GFP reporter mice expressing GFP under control of the motor neuron-specific homeodomain transcription factor HB9 promoter^19^ were crossed with SOD1^G93A^ mice to generate HB9:GFP controls (henceforth called WT) and SOD1^G93A^; HB9:GFP mice (henceforth called SOD1^G93A^). Lumbar spinal cords were dissected, pooled within genotypes, dissociated into a single-cell suspension and GFP+ motor neurons isolated using FACS (Fig. 1A). The percentage of GFP+ cells isolated from WT and SOD1^G93A^ embryos was 15 ± 2.6 and 15 ± 2.2% (mean ± SEM), respectively, when gated against GFP− cells from spinal cord (Fig. 1B).

To confirm the enrichment of motor neurons in the GFP+ cell fraction, high integrity RNA (RNA integrity number >9) was extracted from GFP+, GFP− and unsorted cell populations from WT embryos (Supplementary Fig. 1). Real-time qPCR was conducted to test for selected neuronal and non-neuronal populations. *Mnx1* (*HB9*), the key marker for motor neurons, was significantly enriched (16 ± 3.3-fold) in the GFP+ fraction, relative to the unsorted cell fraction (*P* < 0.0001). The interneuron marker *Ptf1a* was significantly depleted (0.8 ± 0.05-fold) in the positive fraction (*P* < 0.05) and enriched in the negative fraction (1.3 ± 0.4-fold), relative to unsorted cells (*P* < 0.01). No significant change was observed in the expression of *Vglut1* expressed by interneurons. The expression of the oligodendrocyte marker *Mbp* was significantly depleted (1.6 ± 0.1-fold) in both the positive and negative fractions, relative to the unsorted sample (*P* < 0.00001), whereas the astrocytic gene *Gfap* was significantly depleted in the positive fraction (1.0 ± 0.06, *P* < 0.0001) and negative fraction (0.06 ± 0.08, *P* < 0.0001) (Fig. 1C).

To enable unbiased gene expression profiling of motor neurons, RNA sequencing was conducted on GFP+ cells sorted from WT controls and SOD1^G93A^ embryos. Whole transcriptome cDNA libraries were subject to 150 bp paired-end sequencing at an average read depth of 60 million reads per sample. Motor neurons isolated from SOD1^G93A^ embryos showed a 30,000 ± 3,000-fold increase in human *SOD1* expression, compared with WT (*P* < 0.0001) as expected (Fig. 1D–E). Consistent with the qPCR findings, normalized RNA-seq reads showed an enrichment of motor neuron-specific transcripts *Snp25* (3237 ± 119-fold), *Islet1* (1651 ± 136-fold) and *Mnx1* (3713 ± 279-fold). A corresponding depletion of transcripts specific for microglia, oligodendrocytes, Schwann cells, astrocytes and vascular endothelial cells was also detected (Fig. 1F). Following validation of sample purity, examination of the global gene expression profile by PCA showed the samples segregated into two distinct clusters corresponding to genotypes (Fig. 1G), indicating a subtle difference in the motor neuron transcriptome between WT and SOD1^G93A^ mice at E12.5.

### Identification of potential mechanisms involved in susceptibility to ALS

**Figure 2:**
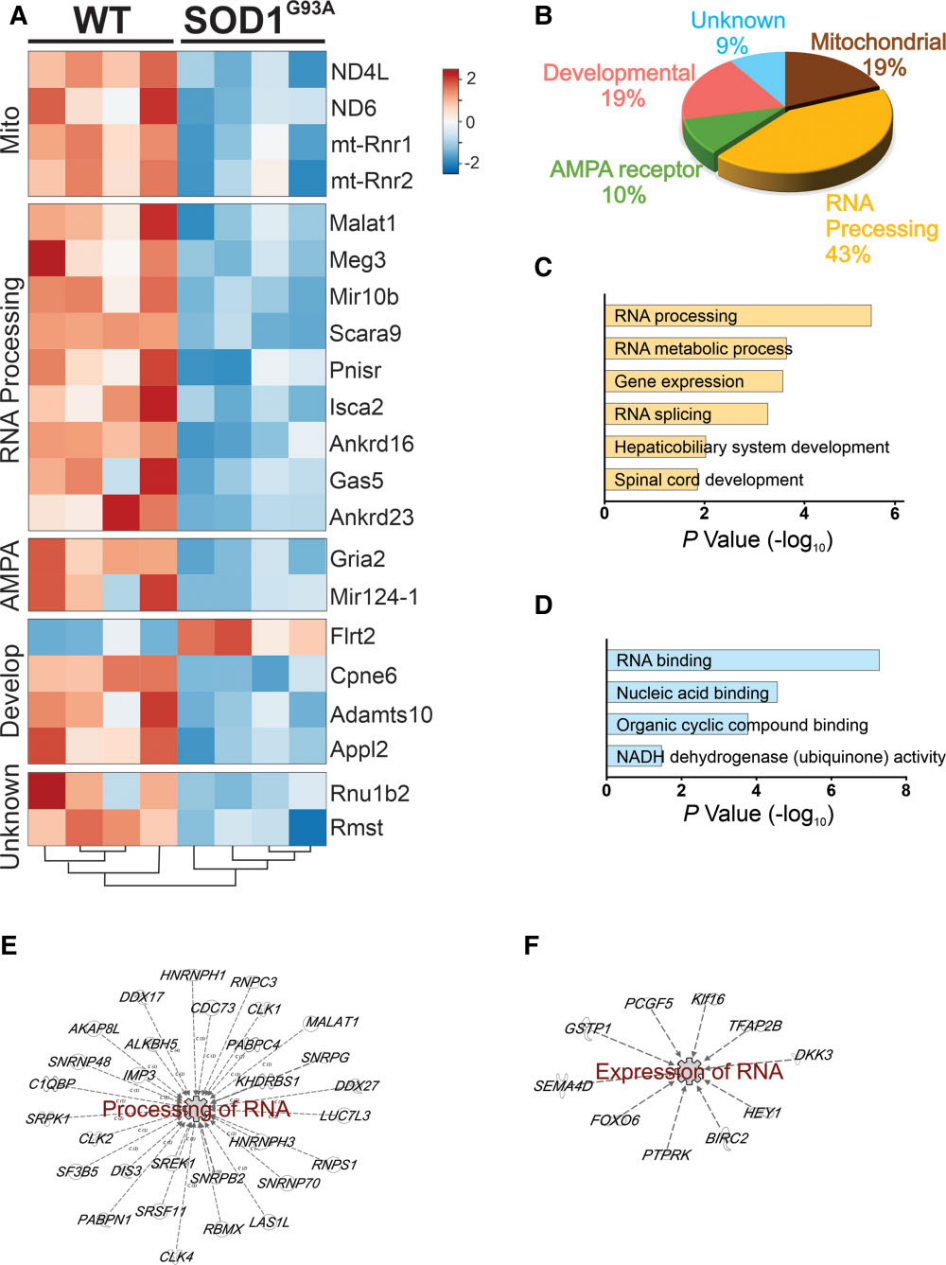
**Deconstructing dysregulated pathways of early pathogenesis in ALS.** (**A**) Heat map shows the relative expression levels of differentially expressed genes, along with the hierarchical clustering of genes and samples. Expression levels used in the heatmap were calculated by mean of the log_2_ normalized values of genes. Visual representation of downregulation and upregulation is shown. (**B**) Classification of the differentially expressed genes based on their known molecular function. (**C**) Enriched biological function and (**D**) molecular function identified by gene ontology analysis (DAVID) using significantly differentially expressed genes (*P* < 0.01). (**E** and **F**) Network analysis using significantly differentially expressed genes (*P* < 0.01) determined by IPA.

Differential expression analysis was conducted to identify significantly dysregulated genes in motor neurons at E12.5. Significant changes (corrected for multiple testing) in 21 genes were observed at E12.5 in SOD1^G93A^ motor neurons compared with WT (*q* value <0.05) (Table 2). Hierarchical clustering of differentially expressed genes indicated the highest similarity between the replicates of each genotype (Fig. 2A). Classification of the differentially expressed genes based on their known molecular function revealed that the majority of genes were involved in RNA processing (43%). The remaining genes were classified as developmental-related (19%), mitochondrial (19%), AMPA receptor signalling (10%) and unknown genes (9%) (Fig. 2B).

**Table 2:** Significantly differentially expressed genes in SOD1^G93A^ motor neurons at E12.5

Gene symbol	Fold change^a^	Adj *P*-value	Entrez ID
***Rmst***	−0.80	4.76 × 10^−02^	110333
***Gria2***	−0.78	1.78 × 10^−04^	14800
***mt-Rnr2***	−0.76	3.88 × 10^−02^	17725
***Appl2***	−0.75	4.76 × 10^−02^	216190
***Pnisr***	−0.73	3.88 × 10^−02^	66625
***ND6***	−0.72	2.88 × 10^−02^	17722
***Isca2***	−0.72	4.76 × 10^−02^	74316
***mt-Rnr1***	−0.70	2.44 × 10^−04^	17724
***Adamts10***	−0.70	3.44 × 10^−02^	224697
***Mir124-1***	−0.66	3.88 × 10^−02^	268755
***Gas5***	−0.65	1.17 × 10^−02^	14455
***Meg3***	−0.63	2.78 × 10^−05^	17263
***Ankrd16***	−0.62	2.62 × 10^−04^	320816
***Malat1***	−0.59	2.07 × 10^−05^	72289
***Mir10b***	−0.55	3.42 × 10^−03^	387144
***ND4L***	−0.54	2.40 × 10^−09^	17720
***Ankrd23***	−0.48	4.76 × 10^−02^	78321
***Cpne6***	−0.40	3.64 × 10^−02^	12891
***Scarna9***	−0.38	5.85 × 10^−03^	100216535
***Rnu1b2***	−0.34	4.48 × 10^−02^	19845
***Flrt2***	1.54	2.44 × 10^−04^	399558

^a^ Negative value represents downregulation.

Gene ontology analysis for biological and molecular processes using a less stringent significance threshold (not corrected for multiple testing) was conducted to capture a broader set of dysregulated genes and pathways (*P* < 0.01, Supplementary Table 3) and showed highly significant enrichment of RNA processing pathways (Fig. 2C–D). Consistent with gene ontology analysis, network analysis using the enlarged list of differentially expressed genes identified significant pathways centred around processing of RNA (Fig. 2E) and mRNA transcription (expression of RNA; *P* < 0.01) (Fig. 2F).

### AMPA receptor expression is dysregulated in SOD1^G93A^ mouse motor neurons at E12.5

**Figure 3:**
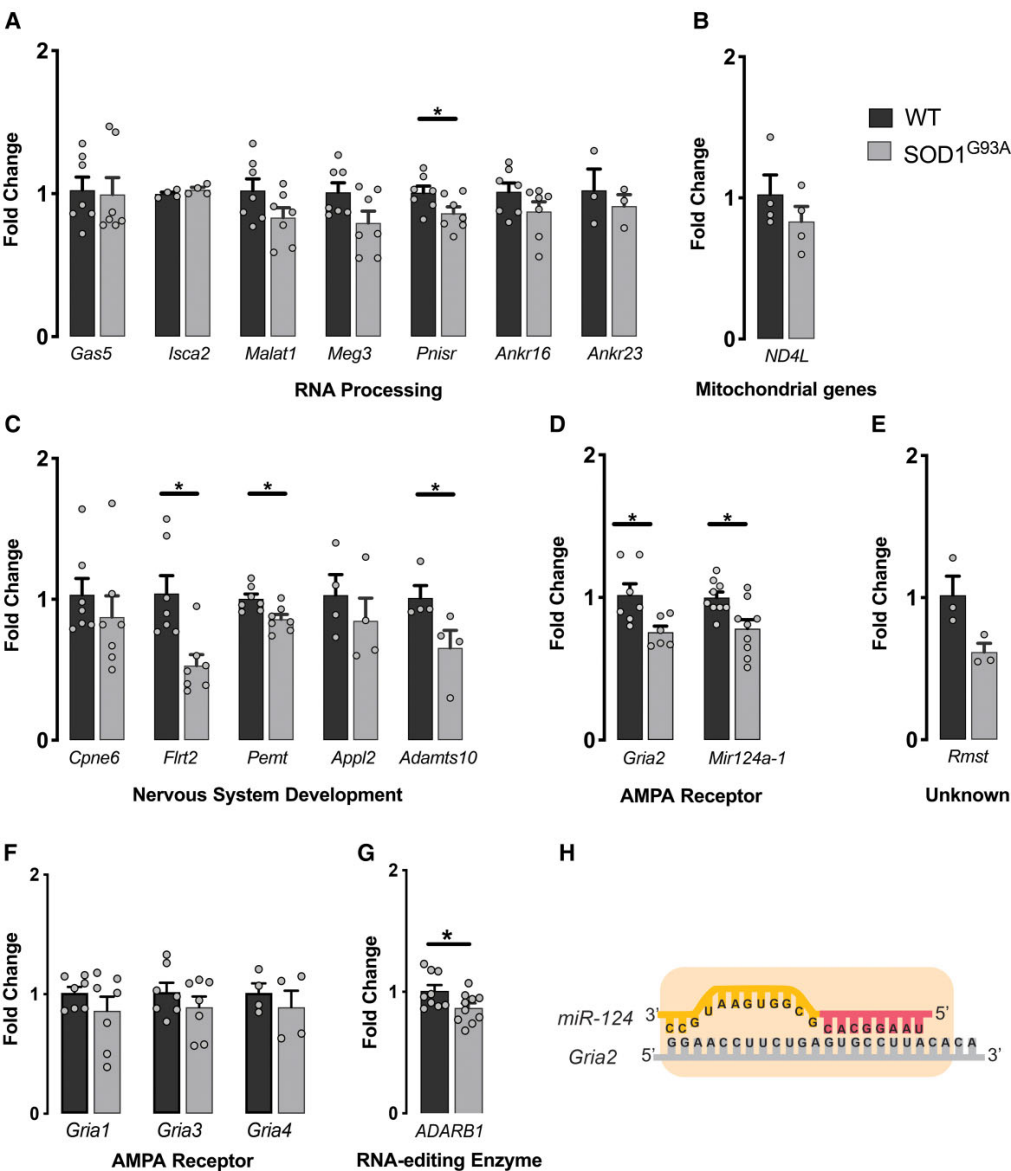
**Validation of differentially expressed genes using qRT-PCR.** (**A–E**) qPCR verification of the expression of genes involved in biological process identified as enriched by GO analysis compared with WT control. **(F)** Fold change expression of Ca^2+^-permeable AMPAR subunit *Gria1*, *Gria3* and *Gria4* mRNAs, relative to WT motor neurons at E12.5. **(G)** Relative expression of *Adarb1* mRNA in SOD1^G93A^ motor neurons at E12.5. (**H**) Schema showing the position of the fully complementary *miR-124* target site at the 5′-end of the mouse *Gria2*, 3′-UTR. The seed region of *miR-124* is shown. Data represent mean ± SEM, unpaired student *t*-test, *n* = 5–7 biological replicates, *P < 0.05.

Validation of significantly changed genes at E12.5 was conducted on a separate batch of GFP+ isolated motor neurons from WT and SOD1^G93A^ mice using real-time qPCR where primers could be designed (16 of 21 genes). A significant decrease in expression was identified in the RNA processing gene *Pnisr* (*−0.2* ± 0.06, *P* = 0.04) (Fig. 3A); no significant changes in the mitochondrial gene was validated (Fig. 3B); nervous system development-related genes *Flrt2* (*−0.5* ± 0.1, *P* = 0.003), *Pemt* (*−0.1* ± 0.04, *P* = 0.01) and *Adamts10* (*−0.2* ± 0.06, *P* = 0.02) (Fig. 3C) and the AMPA receptor gene *Gria2* (*GluA2*; *−0.2* ± 0.09, *P* = 0.01); related *Mir124-1* (*−0.2* ± 0.07, *P* = 0.03) (Fig. 3D), and no significant changes in unknown gene category were identified (Fig. 3E).

*Gria2* encodes GluA2 which reduces Ca^2+^ permeability of AMPA receptors when incorporated within the heteromeric receptor. Deficiency of the GluA2 Subunit of AMPA receptor in motor neurons is associated with Ca^2+^-mediated excitotoxity in ALS.^30^ As excitotoxicity is one of the most important processes implicated in ALS pathogenesis, *Gria2* was selected as an interesting target for further validation. Evidence of mRNA dysregulation in other components of AMPA receptors was then examined in SOD1^G93A^ mouse motor neurons. No significant changes in expression of other AMPA receptor subunits *Gria1*, *Gria3* or *Gria4* were detected at E12.5 (Fig. 3F). However, a significant decrease in the expression of the *Gria2* editing enzyme *Adar2* (*Adarb1*) gene (*0.13* ± 0.06, *P* = 0.01) was identified using qRT-PCR (Fig. 3G).

Interestingly, *Gria2* was shown as a potential target of transcriptional regulation by the significantly changed RNA processing gene *miR124-1* in our study (by Targetscan and miRanda). The 3′-UTR of *Gria2* contains a highly conserved *miR-12-1* target site (Fig. 3H), and it has been previously shown that *miR-124*-mediated regulation of *Gria2* produces rapid changes in *Gria2* expression.^31,32^

To further confirm the involvement of a potential *Gria2* and *Adar2* network, droplet digital PCR was used. A significant downregulation of *Gria2* (19 ± 6 copies per μl, *P* < 0.01) and *Adar2* (3 ± 1.3 copies per μl, *P* < 0.01) transcript numbers were identified in SOD1^G93A^ motor neurons compared with WT (Supplementary Fig. 2A–C). Enzymatic editing of *Gria2* transcripts by *Adar2* regulates its permeability to Ca^2+^ influx. *Gria2* has been reported to be less efficiently edited in motor neurons due to downregulation of *Adar2* in symptomatic SALS patients and mouse models.^33,34^ Hence, RFLP PCR was used to detect *Gria2* mRNA post-transcriptional editing using a Bbv1 endonuclease in SOD1^G93A^ motor neurons at E12.5.^35^ Despite downregulation of *Adar2*, SOD1^G93A^ motor neurons exhibited normal *Gria2* editing at E12.5 (Supplementary Fig. 2D).

### GluA2 is downregulated in motor neurons of embryonic SOD1^G93A^ mice

**Figure 4:**
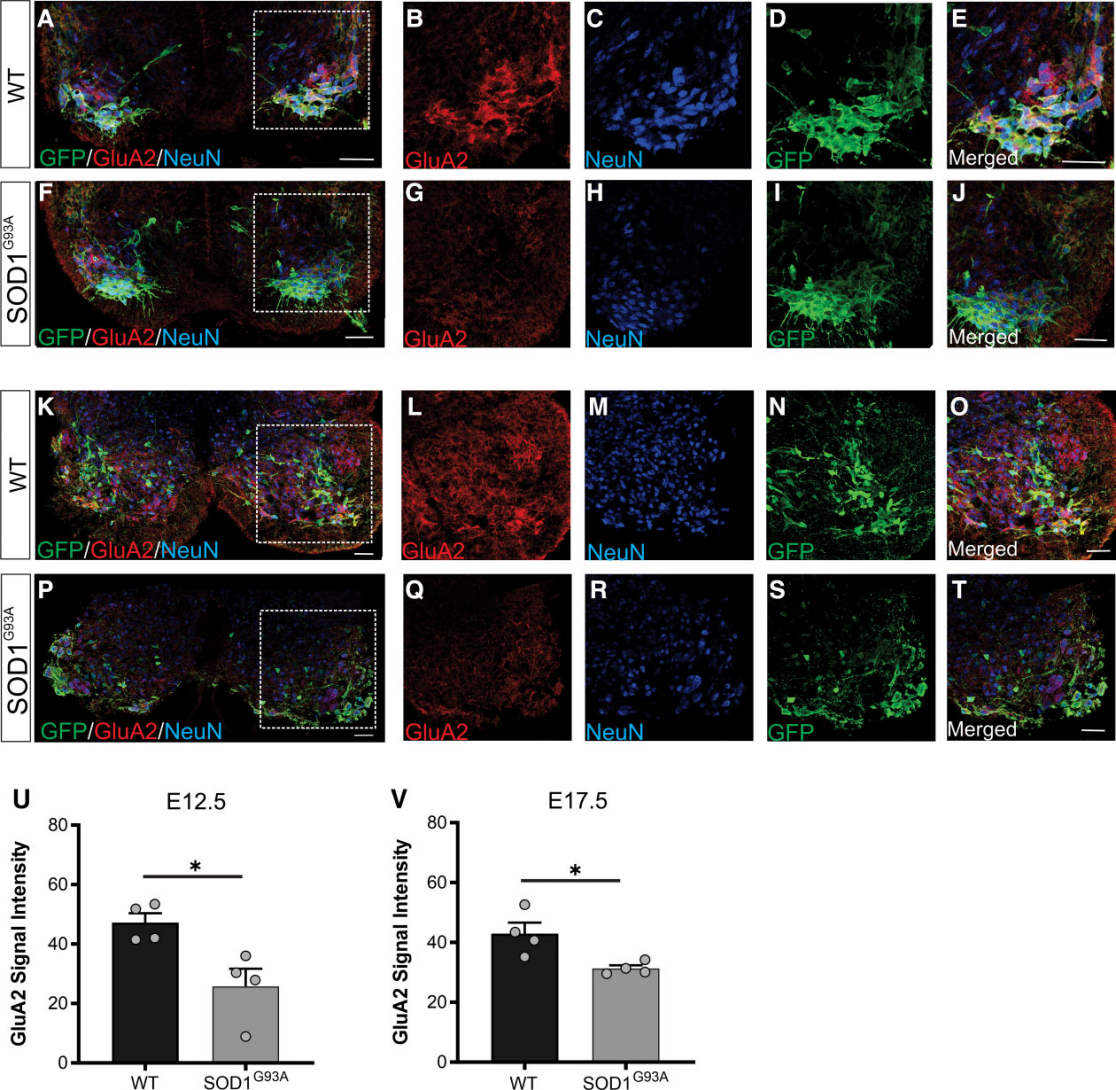
**Expression of GluA2 in spinal cords of embryonic SOD1^G93A^ mice.** Cross-sections of lumbar spinal cord from WT (HB9:GFP; WT) and SOD1^G93A^ (SOD1^G93A^; HB9:GFP) mice at **(A–J)** E12.5 and (**K–T**) E17.5. Double-immunolabelling for GFP, GluA2 and NeuN (Neuronal nuclei). Plots represent quantification analysis of GluA2 signal intensity in HB9:GFP motor neurons at (**U**) E12.5 and (**V**) E17.5. Data represent mean ± SEM, unpaired student *t*-test performed on *n* = 4 biological replicates, ∼50 neurons analysed per biological replicate, *P < 0.05. Scale bars 50 μm.

To determine if GluA2 protein was reduced in motor neurons of SOD1^G93A^ mice, embryonic spinal cords were subjected to immunohistochemistry at E12.5 (Fig. 4A–J) and E17.5 (Fig. 4K–T). GluA2 levels were significantly reduced in GFP + lumbar motor neurons in SOD1^G93A^ mice, compared with WT motor neurons at E12.5 (*P* < 0.05) (Fig. 4U). Examination of E17.5 spinal cord showed that this significant reduction in GluA2 protein levels persisted throughout embryonic development (*P* < 0.05) (Fig. 4V). Furthermore, GFP intensity was not reduced in spinal cords at E12.5 or E17.5 and there was no loss of motor neurons at E17.5 (Supplementary Fig. 3), confirming GluA2 downmodulation in motor neurons. In addition, GluA2 signal was not downregulated in GFP-NeuN+ cells, suggesting a motor neuron-specific property (Supplementary Fig. 3). Thus, GluA2 downregulation is a very early and sustained event in vulnerable motor neurons from SOD1^G93A^ mice.

### AMPA receptor subunit dysregulation occurs in ALS-linked SOD1 patient iPSC-derived motor neurons

**Figure 5:**
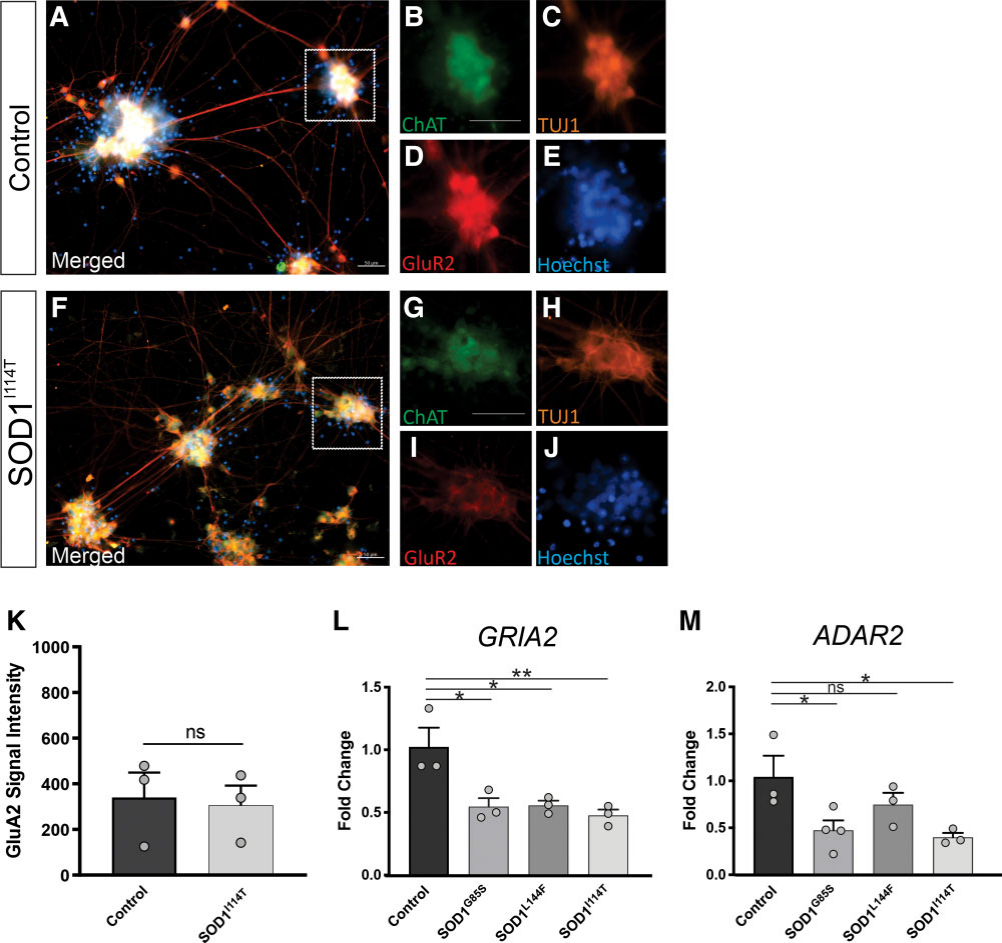
**Expression of *GRIA2* and *ADAR2* in iPSC motor neurons derived from ALS patients with SOD1 mutations and healthy control lines.** Representative images of iPSC mature motor neurons derived from (**A–E**) healthy control line and (**F–J**) SOD1^I114T^ line, immunolabelled with ChAT, GluA2 and TUJ1, counterstained with Hoechst. (**K**) Plot represents quantification analysis of GluA2 signal intensity in iPSC motor neurons. Data represent mean ± SEM, unpaired student *t*-test performed on *n* = 3 biological replicates, 50 neurons analysed per biological replicate. **(L)** Fold change expression of *GRIA2* in SOD1 lines, compared with healthy control line determined by qRT-PCR. (**M**) Fold change expression of *ADAR2* in SOD1 lines, compared with healthy control line determined by qRT-PCR. Data represent mean ± SEM, *n* = 3 biological replicates, one-way ANOVA with Dunnett's multiple comparison test, *P < 0.01, **P < 0.005. Scale bars 50 μm.

To confirm our findings from the SOD1^G93A^ mouse model, we cultured motor neurons from SOD1-linked ALS patient iPSCs. We used lines carrying different SOD1 point mutations generated in-house or previously established.^26^ In 28-day-old motor neurons, there was a trend for reduced GluA2 levels in mutant SOD1 motor neurons compared with control cells by immunocytochemistry, although not statistically significant (Fig. 5A–K), most likely due to the variation in differentiated human motor neurons, in contrast to motor neurons isolated from transgenic SOD1^G93A^ mice which are more homogeneous. However, *GRIA2* expression assessed by qPCR was significantly reduced in SOD1^G85S^ (0.5 ± 0.1-fold, *P* < 0.01), SOD1^L144F^ (0.5 ± 0.1-fold, *P* < 0.01) and SOD1^I114T^ (0.6 ± 0.1-fold, *P* < 0.001), compared with control motor neurons (Fig. 5L). A significant reduction in *ADAR2* expression was observed in SOD1^G85S^ (0.6 ± 0.2-fold, *P* < 0.05) and SOD1^I114T^ (0.6 ± 0.2-fold, *P* < 0.05), but not in SOD1^L144F^ motor neurons, compared with control (Fig. 5M). Again, despite downregulation of *ADAR2*, mutant SOD1 motor neurons revealed normal *GRIA2* editing (Supplementary Fig. 4). Hence, dysregulation of AMPA receptor signalling genes occurs in motor neurons of both SOD1-linked ALS patients and mice.

**Figure 6:**
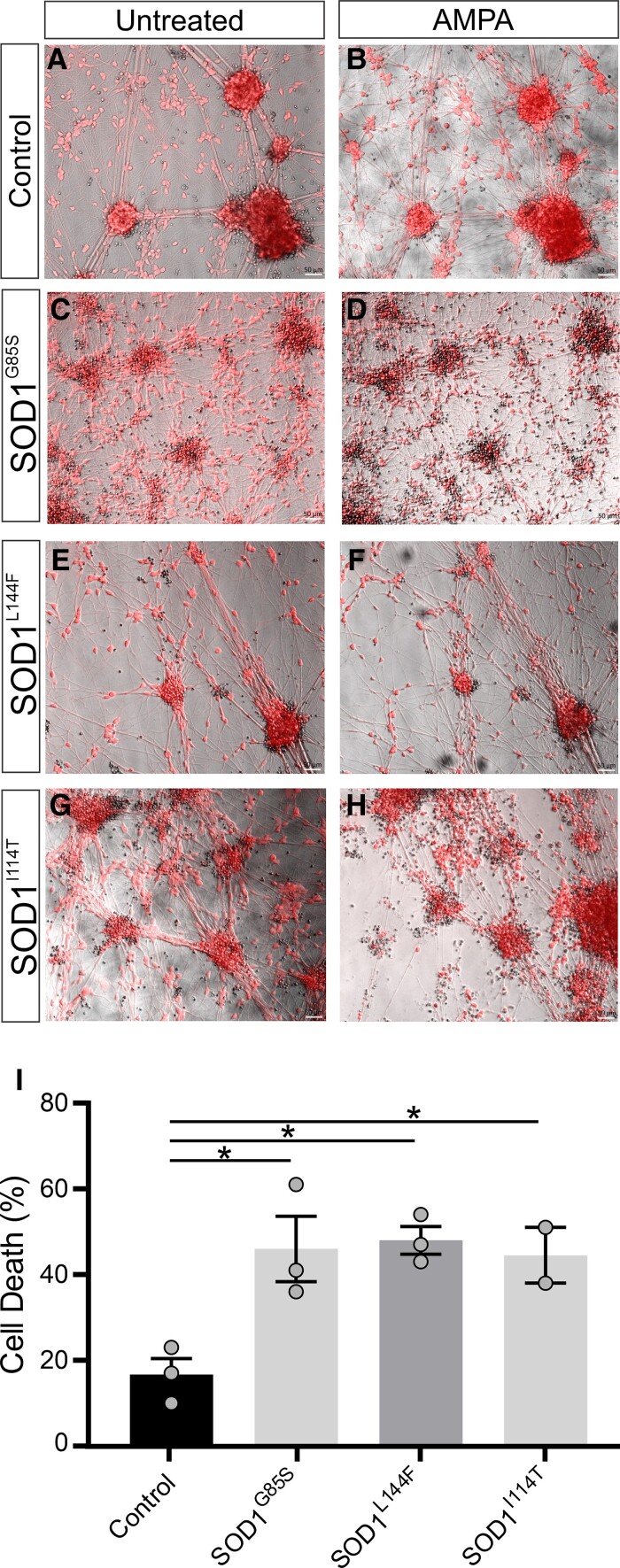
**AMPA-induced excitotoxicity in SOD1 patient-derived motor neurons.** Excitotoxity was induced in Day 28 motor neurons by 100 μM AMPA treatment for 24 h and cell viability was assessed by calcein red staining. Representative images of Calcein Red stained motor neurons derived from (**A–B**) healthy control or (**C–H**) mutant SOD1 lines before and after 24 h treatment with AMPA. (**I**) The bar graph represents percentage of cell death of each line following AMPA treatment. Data represent mean ± SEM, *n* = 3 biological replicates. One-way ANOVA with Dunnett’s multiple comparison test, *P < 0.01. Scale bars 50 μm.

To functionally validate the impact of *GRIA2* downregulation on survival of SOD1 patient-derived motor neurons, we next performed an excitotoxicity assay. Excitotoxicity was induced using the AMPA receptor-selective agonist AMPA. Exposure to AMPA resulted in higher levels of cell death in mutant SOD1 motor neurons (SOD1^G85S^, 46%; SOD1^L144F^, 48% and SOD1^I114T^, 44.5%), compared with control motor neurons (21.6%) (Fig. 6A–I). These findings suggest that SOD1 patient-derived motor neurons show increased vulnerability to AMPA-induced excitotoxicity through reduced *GRIA2* expression which increases Ca^2+^ permeability.

### RNA processing gene dysregulation in ALS-linked SOD1 patient iPSC-derived motor neurons

**Figure 7:**
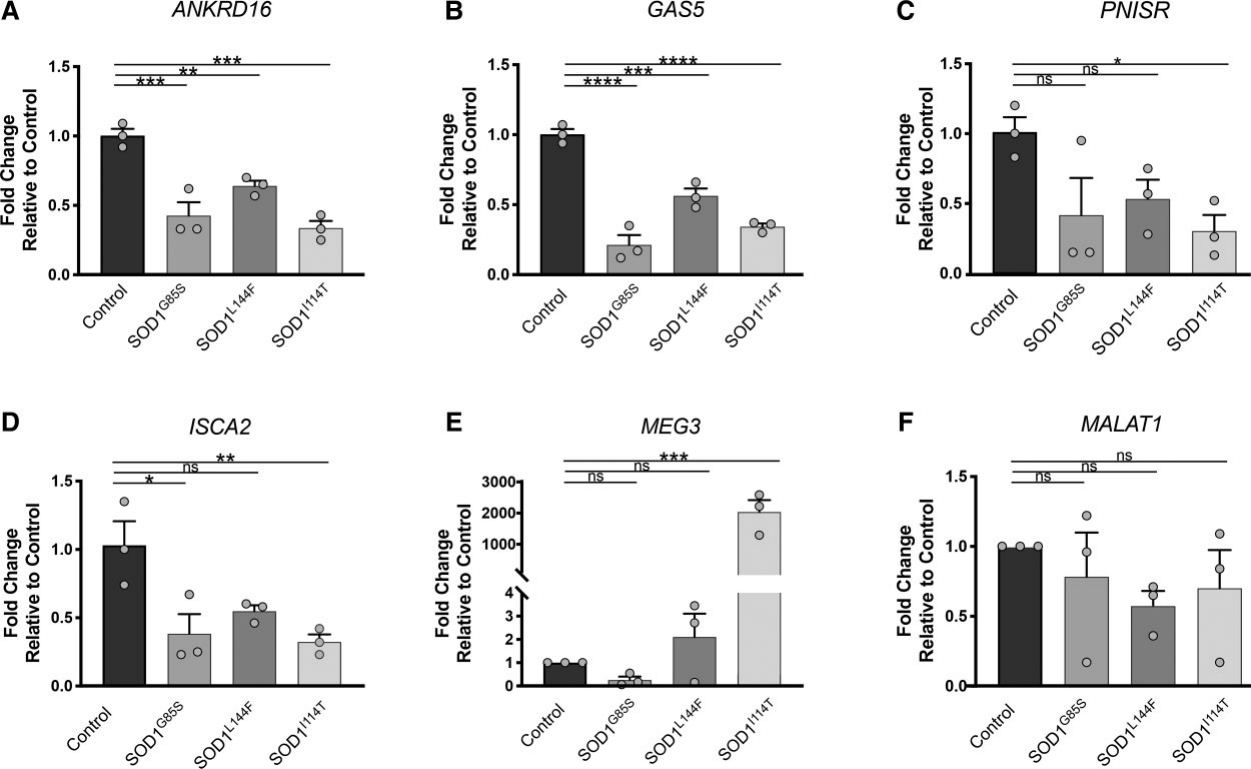
**Expression of RNA processing genes in iPSC motor neurons derived from ALS patients with SOD1 mutations and healthy control lines.** Fold change expression of (**A**) *ANKRD16*, (**B**) *GAS5*, (**C**) *PNISR*, (**D**) *ISCA2*, (**E**) *MEG3* and (**F**) *MALAT1* in SOD1 mutant lines, compared with healthy control line determined by qRT-PCR. Data represent mean ± SEM, *n* = 3 biological replicates. One-way ANOVA with Dunnett’s multiple comparison test, *P < 0.01, **P < 0.005, ***P < 0.0005, ****P < 0.0001. ns, not significant.

Dysregulated RNA processing genes were validated using qPCR in motor neurons from SOD1-linked ALS patient iPSCs. The expression of *ANKRD16* and *GAS5* genes was significantly reduced in SOD1^G85S^ (0.5 ± 0.08-fold, *P* < 0.0005, 0.7 ± 0.1-fold, *P* < 0.0001, respectively), SOD1^L144F^ (0.3 ± 0.08-fold, *P* < 0.009, 0.4 ± 0.06-fold, *P* < 0.0006, respectively) and SOD1^I114T^ (0.6 ± 0.08-fold, *P* < 0.0002, 0.6 ± 0.06-fold, *P* < 0.0001, respectively), compared with control motor neurons (Fig. 7A–B). *PNISR* was downregulated in SOD1^I114T^ (0.7 ± 0.2-fold, *P* < 0.04) but not in SOD1^G85S^ and SOD1^L144F^ motor neurons (Fig. 7C). A significant reduction in *ISCA2* expression was also observed in SOD1^G85S^ (0.6 ± 0.1-fold, *P* < 0.01) and SOD1^I114T^ (0.7 ± 0.1-fold, *P* < 0.01) but not in SOD1^L144F^ motor neurons (Fig. 7D). The expression of *MEG3* was highly elevated in SOD1^I114T^ (2036 ± 271-fold, *P* < 0.0002) but was not changed in SOD1^G85S^ and SOD1^L144F^ motor neurons (Fig. 7E). Finally, *MALAT1* expression was not affected in mutant SOD1 lines, compared with control (Fig. 7F). Thus, dysregulation of RNA processing genes in motor neurons of SOD1^G93A^ mice also occurs in SOD1-linked ALS patients.

## Discussion

Gene expression profiling studies of mouse models of ALS have focussed largely on adult symptomatic and presymptomatic^16–18^ or early postnatal ages.^12^ Although these studies provide molecular insights into potential mechanisms of motor neuron dysfunction and degeneration, they lack resolution of early molecular mechanisms that confer cell vulnerability and are likely to precede cell dysfunction. In this study, using a novel approach with a HB9:GFP reporter mouse, FACS and RNA sequencing, we have characterized the molecular signature of motor neurons from embryonic SOD1^G93A^ mice. The approach we have used has several key advantages over previous gene expression profiling studies in ALS: (i) we have analysed gene expression changes specifically in motor neurons without glial contamination; (ii) we have assessed fetal gene expression changes and (iii) RNA sequencing allows unbiased screening of gene expression in SOD1^G93A^ motor neurons.

To our knowledge, we present the first evidence for motor neuron gene dysregulation at E12.5 in a mouse model of ALS. This is a critical test of the hypothesis proposed by Eisen *et al*.^13^ and our findings provide support for motor neuron transcriptional dysregulation very early in the ALS pathogenic cascade. In a longitudinal microarray study by Saxena *et al*.^12^, ER stress markers and the unfolded protein response (UPR) in SOD1^G93A^ mice were upregulated from P5. Our transcriptomic data reveal no dysregulation of genes involved in ER stress or protein misfolding management pathways, supporting the hypothesis that these pathways are in fact secondary events in ALS pathogenesis. This is corroborated by recent findings that genetic ablation of the key UPR sensor, PERK and downstream mediators GADD34 or CHOP, does not ameliorate the phenotype of SOD1^G93A^ mice.^36^ Results from our study showed that most of the significantly changed genes (*q* < 0.05) were involved in RNA processing, followed by development-related genes, mitochondrial genes and AMPA receptor signalling.

The key finding of the present study was the downregulation of GluA2 subunit of AMPA receptors, at both transcript and protein levels, in embryonic motor neurons of SOD1^G93A^ mice. Decreased expression of GluA2 may potentially confer selective cell vulnerability by allowing Ca^2+^ influx into motor neurons, leading to glutamate-mediated excitotoxicity,^37^ a proposed central pathogenic mechanism and treatment target in ALS. This was confirmed by the increased susceptibility of mutant SOD1 ALS patient iPSC-derived motor neurons to AMPA-induced excitotoxicity, which also demonstrated GluA2 downregulation. Our finding of dysregulated AMPA receptor subunit expression in SOD1-linked ALS patient-derived motor neurons is also consistent with motor neurons from C9orf72-linked ALS patients,^38^ suggesting a common early disease pathway in ALS. Indeed, genetic GluA2 deficiency or overexpression strongly modifies disease progression in SOD1^G93A^ mice.^37,39^ Further, GluA2 deficiency promotes mutant SOD1 misfolding and aggregation in spinal cords of SOD1^G93A^ mice^39^ and our findings place GluA2 dysregulation upstream of mutant SOD1 aggregation and cellular homeostatic responses, such as ER stress. Finally, riluzole which attenuates excitotoxicity remains the forefront approved drug for ALS, arguing for the primacy of excitotoxicity in ALS pathophysiology.

The reduced Ca^2+^ permeability of GluA2-containing AMPA receptors is due to the presence of arginine at position 586 (Q/R site) of the GluA2 protein, instead of the genetically encoded glutamine.^40^ This arginine residue at the Q/R site is introduced by the editing of *Gria2* pre-mRNA, which is mediated by adenosine deaminase acting on RNA 2 (*Adar2*) and is critical to the survival of motor neurons.^41,42^ Here, we identified downregulation of *Adar2*, an enzyme that edits *Gria2* transcripts, in motor neurons of SOD1^G93A^ mice, although we did not detect inefficient editing of *Gria2* in spinal motor neurons of embryonic SOD1^G93A^ mice. Downregulation of *ADAR2* and inefficient *Gria2* RNA editing at Q/R site have been shown to occur in motor neurons of almost all ALS patients.^43–45^ However, RNA editing deficiencies at the Q/R site were not observed in SOD1^G93A^ mice,^46^ concordant with our findings. Interestingly, selective *Adar2* knockout in motor neurons leads to ALS-like symptoms and pathology in mice,^47^ supporting both *Gria2* and *Adar2* as susceptibility factors for motor neurons. Our study significantly advances these findings by placing *Gria2* and *Adar2* perturbations in motor neurons very early in the pathogenic cascade of ALS. We also demonstrated *Gria2* and *Adar2* transcript downregulation in iPSC-derived motor neurons from ALS patient carrying SOD1 point mutations which further validated our findings since these cells have a fetal transcriptome similar to mouse motor neurons at E12.5. Thus, transcriptional dysregulation in SOD1^G93A^ mice shows high concordance with ALS patient-derived motor neurons, supporting the preclinical validity and usefulness of mutant SOD1 mice.

In this study, we also found *Mir-124-1* downregulation in embryonic SOD1^G93A^ motor neurons and computational identification of a conserved *miR-124-1* site in the 3′-UTR of *Gria2* gave rise to the hypothesis that *Mir-124*-mediated regulation of *Gria2* could produce rapid changes in GluA2 expression. However, our results conflict with previous published reports of an inverse correlation of *Mir124-1* and *Gria2* levels.^48^ How *Mir-124* regulates the expression of *Gria2* in motor neurons and the exact molecular link to mutant SOD1 remains to be elucidated.

A novel finding of our study was dysregulation of RNA processing genes, particularly *Pnisr*, in mutant SOD1 motor neurons at E12.5 as confirmed by qPCR. Further studies are required to uncover the exact functional role and the downstream effect of *Pnisr* dysregulation in SOD1^G93A^ mice. Dysregulation of RNA processing genes was further validated using qPCR in ALS patient iPSC-derived motor neurons. A significant downregulation of *ANKRD16*, *GAS5, PNISR* and *ISCA2* genes were identified in SOD1 lines relative to controls, consistent with RNA-seq findings. RNA dysregulation is a key contributor to ALS pathogenesis. The major ALS-causative gene mutations in TDP-43, FUS and C9orf72 are involved in aspects of RNA metabolism processes, such as mRNA transcription, alternative splicing, RNA transport, mRNA stabilization and miRNA biogenesis.^49^ Evidence of RNA processing dysregulation in presymptomatic SOD1^G93A^ mice has not been shown previously; however, using a combination of laser-capture microdissection and microarray analysis, downregulation of mRNA processing genes in SOD1^G93A^ motor neurons at P90 was identified.^18^ Our findings, therefore, support an early and key role of RNA processing gene dysregulation in SOD1-linked ALS.

**Figure 8:**
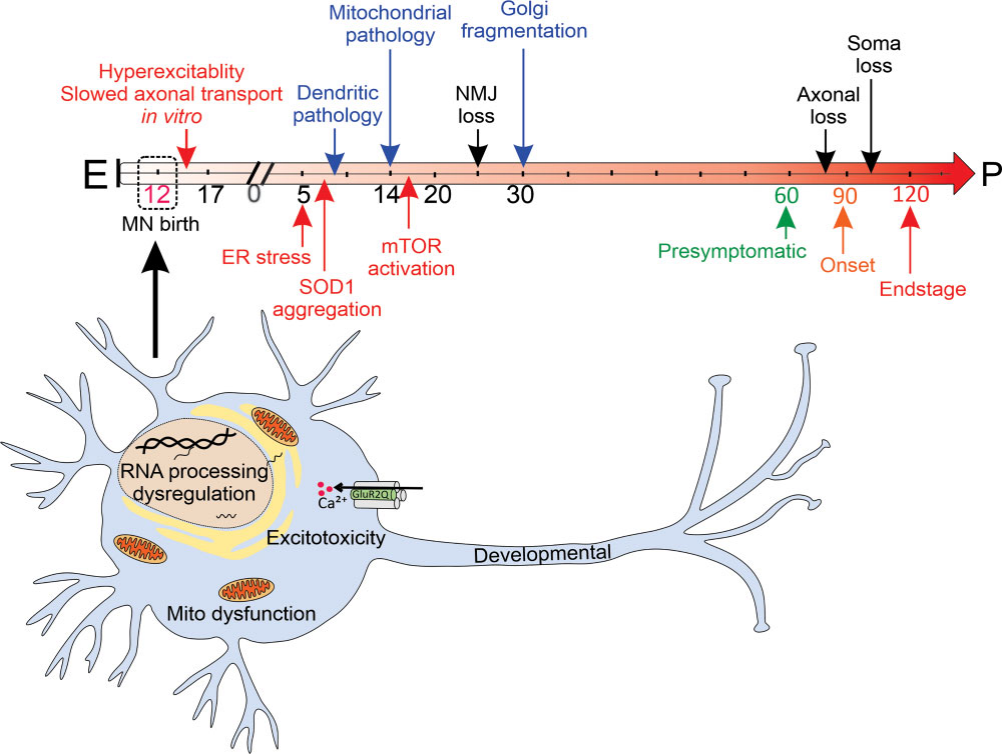
**Temporal sequence of major dysregulated cellular mechanisms in spinal motor neurons of SOD1^G93A^ mice.** In SOD1^G93A^ mice, the onset of clinical symptoms occurs at postnatal (P) Day 90. The key pathological, subcellular and molecular events previously reported are summarized in the above timeline. In this study, we identified the earliest known dysregulated mechanisms as shown at embryonic (E) Day 12.5 that may confer motor neuron vulnerability, involving dysfunction of RNA processing, mitochondria and AMPA receptor-mediated excitotoxicity. Mito, mitochondria; NMJ, neuromuscular junction.

In summary, this study provides the earliest evidence of transcriptional dysregulation occurring in affected motor neurons of SOD1^G93A^ mice *in utero*, supporting a potential perinatal origin of ALS as proposed by others.^13^ Our data highlight dysregulation of AMPA receptor Ca^2+^ permeability and RNA processing as very early and intrinsic events in the pathogenic cascade of mutant SOD1 (Fig. 8). Based on our evidence for AMPA receptor dysregulation in embryonic motor neurons of SOD1^G93A^ mice and SOD1-linked ALS patient-derived motor neurons, we propose that aberrant Ca^2+^-mediated influx and signalling are proximal and key events, leading to downstream consequences such as hyperexcitability, excitotoxicity, ER stress and protein aggregation which may account for selective motor neuron death in ALS.

## Supplementary Material

fcac081_Supplementary_Data

## Abbreviations

ADAR2 =: adenosine deaminase acting on RNA 2
ALS =: amyotrophic lateral sclerosis
AMPA =: α-amino-3-hydroxyl-5-methyl-4-isoxazole-propionate
E =: embryonic day
ER =: endoplasmic reticulum
FACS =: fluorescence-activated cell sorting
GluA2 =: AMPA receptor 2 subunit
GFP =: green fluorescent protein
Gria2 =: glutamate ionotropic receptor AMPA type Subunit 2
HB9 =: homeobox HB9
iPSC =: induced pluripotent stem cell
mito =: mitochondrial
P =: postnatal day
PCA =: principal component analysis
RFLP =: restriction fragment length polymorphism
SEM =: standard error of the mean
SOD1 =: superoxide dismutase 1
WT =: wild-type
