# Supplementary material for: α-Amino-3-hydroxyl-5-methyl-4-isoxazole-propionate receptor and RNA processing gene dysregulation are early determinants of selective motor neuron vulnerability in a mouse model of amyotrophic lateral sclerosis
Source: Brain Commun. 2022 Mar 31;4(2):fcac081. doi: 10.1093/braincomms/fcac081 (PMC9016138; doi:10.1093/braincomms/fcac081)
Supplement: fcac081_Supplementary_Data [file fcac081_supplementary_data.pdf]

Supplementary Materials

Material and Methods

Droplet digital PCR

The ddPCR analysis was performed on a QX200 system (Bio-Rad, 1864001). The reaction mixture was in a volume of 20 µL and comprised 1 × ddPCR Supermix for Probes (No dUTP) (Bio-Rad, 1863024), 1 µL of 20x primers mixture and probe labelled with FAM (IDT, cat number) nuclease free water and 1:10 µL of cDNA (50 ng). Droplet generation, PCR and analysis of droplets were conducted as as previously described in detail (Hughesman *et al.*, 2016). PCR protocol is listed in **Supplementary Table 1.**

Supplementary Table 1. Cycling conditions for Bio-Rad's C1000 Touch Thermal Cycler.

Cycling step	Temperature(C)	Time	Ramp rate	Number of cycles
**Enzyme activation**	95	10 min		1
**Denaturation**	94	30 sec		40
**Annealing/extension**	60	1 min	2°C/sec	
**Enzyme deactivation**	98	10 min		1
**Hold**	4	Infinite		1

Restriction fragment length polymorphism (RFLP)

Twenty ng of cDNA was used for amplifying a product across the edited base of *Gria2*. The polymerase chain reaction (PCR) was performed using HotStar HiFidelity Polymerase Kit (QIAGEN; 202602) according to manufacture instruction using forward primer 5'-GGTTTTCCTTGGGTGCCTTTAT-3' and reverse primer 5’-ATCCTCAGCACTTTCGATGG-3'. Primers spanned exons 11 and 12 of *Gria2* to ensure products were derived from mRNAs. Amplification product length was 187 bp. The thermal cycling conditions for RFLP are listed in **Supplementary Table 2**.

PCR product was then digested with the restriction enzyme Bbv1 (NE Biolabs; R0173S) according to manufacture instruction. The digested PCR product resolved on a 3% agarose gel (Scientfix; 9010B) and imaged using ChemiDoc^{TM} MP Imaging System (Bio-Rad).

Supplementary Table 2. The thermal cycling condition for Gria2 RFLP.

PCR condition	Temperature (°C)	Time	Cycle
**Initial denaturation**	95	5 min	1
**Denaturation**	94	15 sec	
**Annealing**	60	1 min	35
**Extension**	72	1 min	
**Final extension**	72	10 min	1

Supplementary Figures

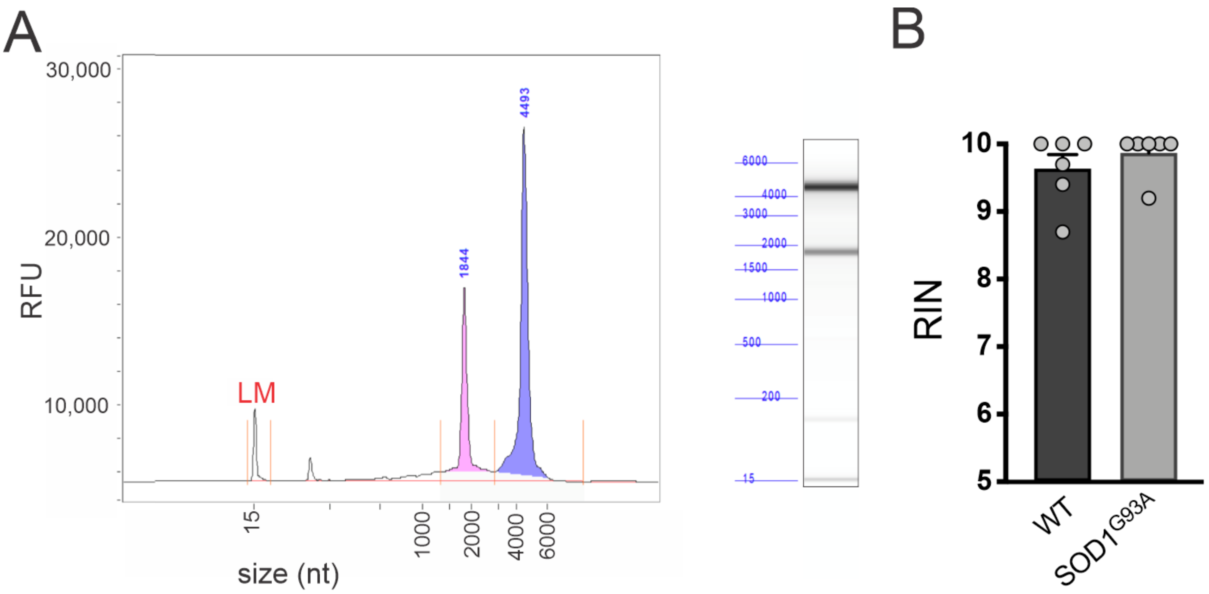

Supplementary Figure 1. Assessment of RNA quantity and integrity of sorted HB9:GFP motor neurons at E12.5.

(**A**) Representative electropherogram traces and corresponding electrophoresis gel images of fragment analyser. Ladder marker (LM) with a 15 nucleotide [nt] molecular weight marker and peaks corresponding to the 18S and 28S ribosomal RNA's (rRNA's) have been labelled accordingly. Fragment lengths were plotted along the x-axis with RNA concentrations defined by arbitrary units of fluorescence [FU] plotted along the y-axis. (**B**) RNA integrity number (RIN) quantification. Data presented as mean ± SEM, *n* = 6 biological replicates.

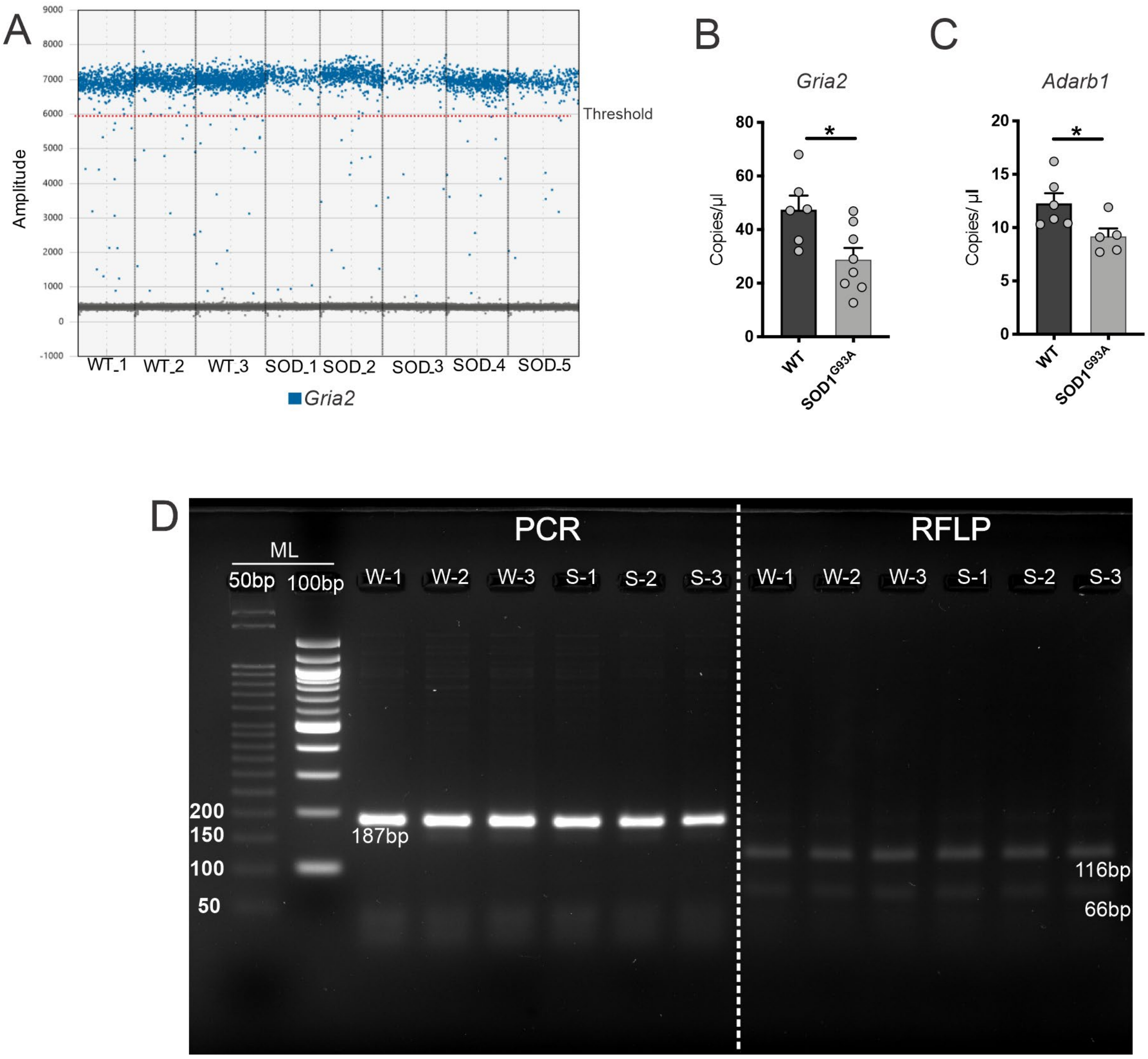

Supplementary Figure 2. Quantification of *Gria2* transcript copies using digital PCR and characterisation of the editing efficiency of *Gria2* in SOD1^{G93A} motor neurons at E12.5.

(A) Representative scatter plot showing copy number analysis by droplet digital PCR of *Gria2*. Blue dots indicate droplets with a fluorescence amplitude above (positives) and grey dots indicate droplets with a fluorescence amplitude below (negatives) the threshold. Positive droplets indicate the presence of target within the droplet, in which end-point PCR produced an amplicon. The ratio of the positive to the total number of droplets allows for calculation of the number of copies of *Gria2* (**B**) Quantification of *Gria2* and (**C**) *Adarb1* transcript copies per µl. (**D**) Representative gel images showing *Gria2* Q→R RNA editing efficiency in SOD1^{G93A} and wildtype motor neurons at E12.5. Left image indicates PCR product of *Gria2*. Right image represents restriction fragment length polymorphism (RFLP) analysis, where efficient *Gria2* RNA editing results in RFLP amplicons of 116 and 66 bp. A band at 81 bp would be observed if *Gria2* subunit is inefficiently edited. Data present as mean ± SEM, n = 3-5 biological replicates. Student t test, * $P < 0.01$. Abbreviations: ML= Molecular Ladder, W= wildtype, S= SOD1^{G93A}.

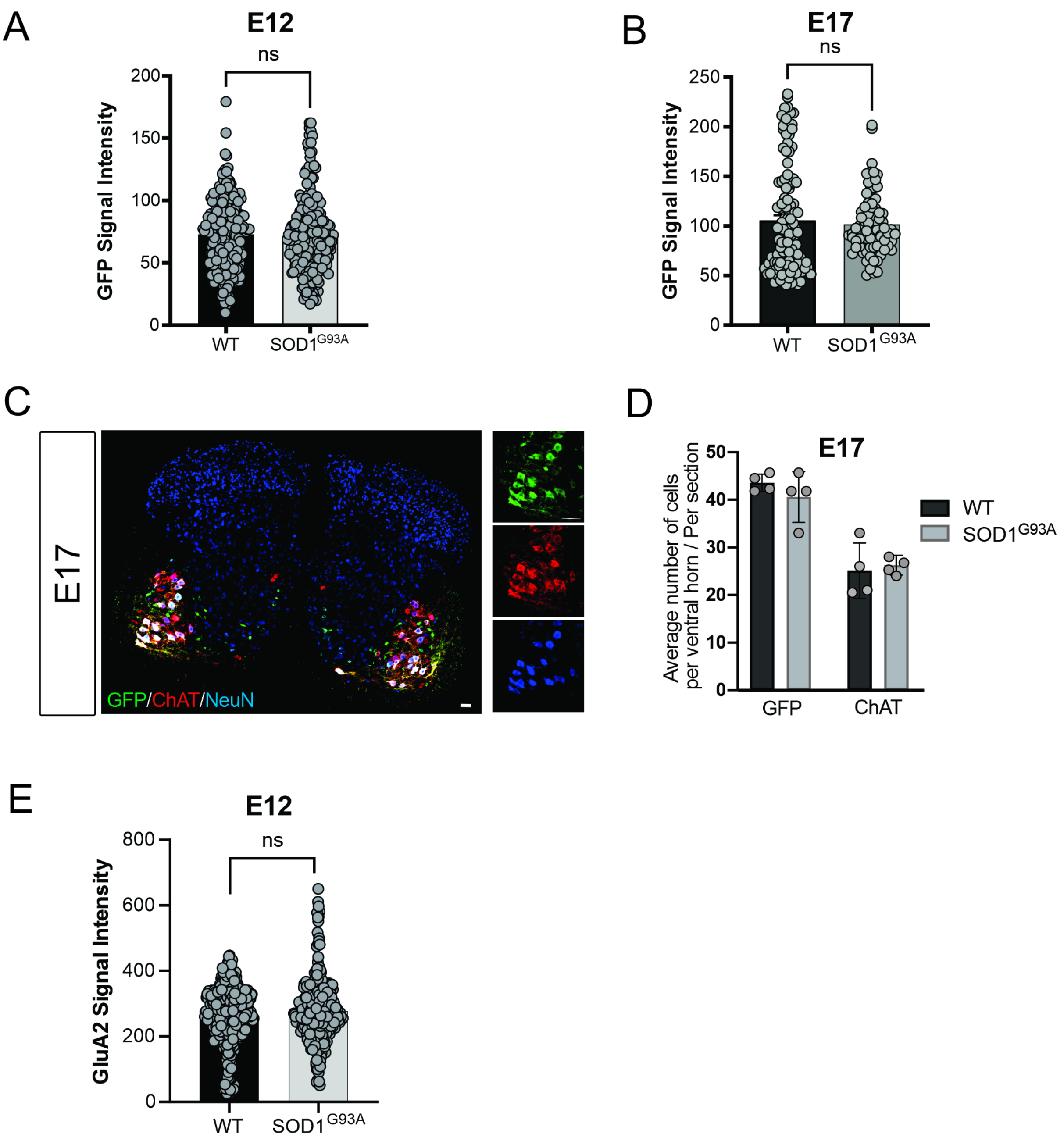

Supplementary Figure 3. GFP signal and motor neuron counts in spinal cords of E12.5 and E17.5 mice.

Plots represent quantification analysis of GFP signal intensity in (A) E12.5 and (B) E17.5 HB9:GFP motor neurons. *n* = ~200 neurons from 4 biological replicates, unpaired student t test. (C) Cross-section of lumbar spinal cord from wild-type (HB9:GFP; WT) mouse at E17.5. Double-immunolabelling for GFP (green), ChAT (red) and NeuN (neuronal nuclei; blue). Scale bar 50 μm. (D) Quantification of GFP+ and ChAT+ cells from spinal cords of mice at E17.5. n = 4 biological replicates, one-way ANOVA. (E) Quantification of GluA2 signal intensity in E12.5 GFP-NeuN+ cells. *n* = ~200 neurons from 4 biological replicates, unpaired student t test.

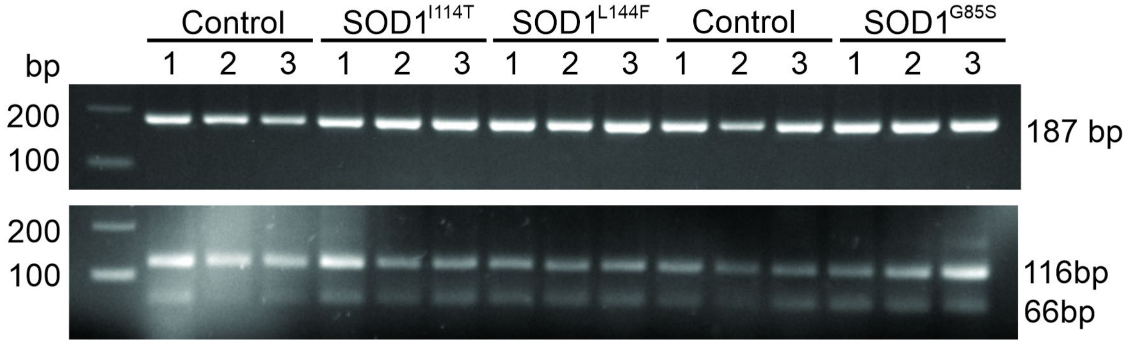

Supplementary Figure 4. Characterisation of the editing efficiency of *GRIA2* in human mutant SOD1 motor neurons.

Representative gel images showing *Gria2* Q→R RNA editing efficiency in control and mutant SOD1 iPSC-derived motor neurons. Top image indicates PCR product of *Gria2*. Bottom image represents restriction fragment length polymorphism (RFLP) analysis, where efficient *Gria2* RNA editing results in RFLP amplicons of 116 and 66 bp. A band at 81 bp would be observed if *Gria2* subunit is inefficiently edited. Data present as mean ± SEM, $n = 3$ biological replicates.

Supplementary Table 3. Extended list of differentially expressed genes at E12.5 in SOD1^{G93A} motor neuron compared to wildtype ($P < 0.01$).

Symbol	Fold change	P value	Entrez ID
ND4L	0.54	1.6x10^-13	17720
Malat1	0.59	2.7x10^-9	72289
Meg3	0.63	5.5x10^-9	17263
Gria2	0.78	4.7x10^-8	14800
mt-Rnr1	0.70	8.4x10^-8	17724
Flrt2	1.54	9.7x10^-8	399558
Ankrd16	0.62	1.2x10^-7	320816
NA	2.39	3.2x10^-7	100862060
NA	2.34	4.5x10^-7	100862044
Mir10b	0.55	2.2x10^-6	387144
Scarna9	0.38	4.2x10^-6	100216535
Gas5	0.65	9.3x10^-6	14455
ND6	0.72	2.4x10^-5	17722
Adamts10	0.70	3.2x10^-5	224697
Cpne6	0.40	3.6x10^-5	12891
mt-Rnr2	0.76	4.2x10^-5	17725
Pnisr	0.73	4.5x10^-5	66625
Mir124-1	0.66	4.6x10^-5	268755
Rnu1b2	0.34	5.6x10^-5	19845
Rmst	0.80	6.8x10^-5	110333
Appl2	0.75	6.9x10^-5	216190
Ankrd23	0.48	7.0x10^-5	78321
Isca2	0.72	7.2x10^-5	74316
NA	0.60	8.1x10^-5	100503377
Mir5125	0.63	9.6x10^-5	100628593
Pxk	0.80	1.1x10^-4	218699
Gm10644	2.38	1.1x10^-4	100126034
Uggt2	0.78	1.2x10^-4	66435
NA	1.99	1.4x10^-4	100862086
Hoxd4	0.72	1.4x10^-4	15436
Miat	0.69	1.6x10^-4	330166
Lrrc45	0.72	1.7x10^-4	217366
Srsf11	0.85	1.9x10^-4	69207

Zfp57	0.72	1.9x10^-4	22715
Cspg4	2.09	2.0x10^-4	121021
NA	4.42	2.2x10^-4	100043126
Pfkp	0.67	2.2x10^-4	56421
Rtel1	0.78	2.3x10^-4	269400
Gm19976	2.04	2.4x10^-4	100503938
Mir1905	2.45	2.5x10^-4	100316808
Brd8	0.82	2.7x10^-4	78656
Gnl3	0.75	2.7x10^-4	30877
NA	2.54	2.8x10^-4	100503773
Gm19383	2.08	3.0x10^-4	100502801
BC051142	0.45	3.4x10^-4	407788
Rnu12	0.55	4.0x10^-4	104307
Gm1821	1.17	4.0x10^-4	218963
Med12	0.83	4.1x10^-4	59024
Snapc4	0.76	4.2x10^-4	227644
Caskin1	0.78	4.2x10^-4	268932
Snord8	0.46	4.3x10^-4	100217445
Leng8	0.72	4.6x10^-4	232798
Mir301	0.44	5.2x10^-4	723834
Firre	0.73	5.8x10^-4	103012
Pnpt1	0.81	5.8x10^-4	71701
Gm11149	3.25	6.1x10^-4	100036537
Degs2	4.53	6.1x10^-4	70059
Cobl	1.38	6.1x10^-4	12808
Pnn	0.82	6.3x10^-4	18949
NA	1.61	6.4x10-4	100861649
NA	0.78	6.7x10^-4	100042464
Trmt13	0.74	6.7x10^-4	229780
Snrnp48	0.74	6.8x10^-4	67797
Gm17275	2.15	7.0x10^-4	100504581
Abcc9	2.11	7.0x10^-4	20928
Tmem176b	1.42	7.2x10^-4	65963
NA	0.62	7.3x10^-4	100628579
Tfap2b	1.34	7.4x10^-4	21419
Snord14d	0.52	7.4x10^-4	100302593

NA	2.24	7.5x10^-4	100503934
Paxbp1	0.76	7.6x10^-4	67367
Fam193b	0.81	8.9x10^-4	212483
NA	1.96	9.3x10^-4	100503527
Fam227a	0.78	9.6x10^-4	75729
Snhg9	0.43	9.8x10^-4	73474
Gpr137b	0.78	1.0x10^-3	83924
NA	1.88	1.0x10^-3	100503512
Arpp21	0.73	1.0x10^-3	74100
4632427E13Rik	0.68	1.1x10^-3	666737
Gm17638	3.15	1.2x10^-3	100504021
Npy	1.32	1.2x10^-3	109648
AA474408	0.48	1.2x10^-3	767812
NA	2.10	1.3x10^-3	100861688
6430411K18Rik	0.58	1.3x10^-3	76880
Snord70	0.51	1.3x10^-3	100217459
Gm16617	33.25	1.3x10^-3	100502764
Mir874	0.03	1.4x10^-3	100124491
Bcas1	2.13	1.4x10^-3	76960
Plekha5	0.85	1.5x10^E-3	109135
Csf2ra	0.68	1.5x10^-3	12982
5031425E22Rik	0.72	1.5x10^-3	269630
Chuk	0.83	1.6x10^-3	12675
Arglu1	0.76	1.6x10^-3	234023
Snord87	0.44	1.6x10^-3	266793
Htra3	3.34	1.6x10^-3	78558
Mir7-1	0.46	1.7x10^-3	723902
Sdccag8	0.80	1.7x10^-3	76816
Hdac10	0.75	1.7x10^-3	170787
NA	1.92	1.7x10^E-3	100862073
Tonsl	0.71	1.7x10^-3	72749
NA	2.63	1.8x10^-3	100861974
Slc25a27	0.81	1.8x10^-3	74011
Rnu73b	0.52	1.9x10^E-3	19871
1600020E01Rik	0.65	1.9x10^-3	72012
AY074887	29.53	1.9x10^-3	246735

Snord110	0.46	2.0x10^-3	100217452
Rfx5	0.75	2.0x10^-3	53970
Gm19544	0.58	2.0x10^-3	100503083
Dgkq	0.78	2.2x10^-3	110524
Gpx2-ps1	0.39	2.2x10^-3	14777
Pcdhgc3	1.18	2.2x10^-3	93706
Gramd1a	0.80	2.3x10^E-3	52857
Pan2	0.84	2.4x10^-3	103135
Dnah17	0.43	2.5x10^-3	69926
Srpk1	0.86	2.5x10^-3	20815
Amfr	1.13	2.7x10^-3	23802
Fam43a	1.33	2.8x10^-3	224093
Cln8	1.22	2.8x10^-3	26889
Trmt6	0.82	2.8x10^-3	66926
NA	2.08	2.8x10^-3	100861900
Mrpl21	0.75	2.9x10^-3	353242
Clcn2	0.76	3.0x10^-3	12724
Dgat1	0.79	3.1x10^-3	13350
Clk2	0.84	3.1x10^-3	12748
Rps28	0.81	3.1x10^-3	54127
Zbtb34	1.16	3.1x10^-3	241311
Snord47	0.53	3.1x10^-3	100217446
Mbip	0.80	3.1x10^-3	217588
Snord55	0.54	3.1x10^-3	100216533
NA	0.29	3.1x10^-3	666890
Zfp810	0.82	3.3x10^-3	235050
Polr3gl	0.74	3.4x10^-3	69870
Ift88	0.81	3.4x10^-3	21821
Igfbp2	1.37	3.4x10^-3	16008
Nrbp2	0.75	3.5x10^-3	223649
Rpl30	0.75	3.5x10^-3	19946
Srek1	0.86	3.6x10^-3	218543
Gm5577	0.67	3.7x10^-3	434064
Ptpn7	11.17	3.8x10^E-3	320139
Stk17b	1.46	3.8x10^-3	98267
Ttc14	0.82	3.9x10^-3	67120

Apoo	0.82	3.9x10^-3	68316
Lbx1	1.54	4.0x10^-3	16814
Mirg	0.65	4.0x10^-3	100040724
Hook2	0.77	4.0x10^E-3	170833
Tatdn3	0.66	4.0x10^-3	68972
NA	1.99	4.1x10^-3	100862399
NA	0.72	4.1x10^-3	100046763
Sorcs2	1.29	4.1x10^-3	81840
Rmnd1	0.78	4.1x10^-3	66084
Srrm2	0.87	4.2x10^-3	75956
E030047D23Rik	0.69	4.3x10^-3	100038605
Fam89a	4.84	4.3x10^-3	69627
Klhl21	1.72	4.6x10^E-3	242785
Hopx	1.74	4.7x10^-3	74318
Rn18s	0.81	4.7x10^-3	19791
Ptprk	0.85	4.7x10^-3	19272
Paqr9	1.43	4.8x10^-3	75552
Rnu11	0.45	4.8x10^-3	353373
Atg16l1	0.82	4.8x10^-3	77040
Chrm2	1.61	4.9x10^-3	243764
Spata1	0.64	5.1x10^-3	70951
Sft2d1	0.77	5.1x10^-3	106489
Pdlim7	0.75	5.3x10^-3	67399
Las1l	0.81	5.3x10^-3	76130
NA	0.66	5.4x10^-3	100504017
Snhg11	0.63	5.4x10^-3	319317
Gm16059	2.07	5.4x10^-3	100503109
Vps16	0.84	5.4x10^-3	80743
Gpd2	1.27	5.5x10^-3	14571
Rps24	0.82	5.6x10^-3	20088
Snord65	0.55	5.6x10^-3	100217444
Tia1	0.86	5.6x10^-3	21841
Vwa5b2	0.72	5.6x10^-3	328643
Snora16a	0.47	5.7x10^-3	100310813
Rab4a	1.21	5.7x10^-3	19341
Smoc1	1.27	5.7x10^-3	64075

Pmaip1	2.52	5.8x10^-3	58801
Zfp207	0.88	5.9x10^-3	22680
Slc25a32	1.40	5.9x10^-3	69906
Pet100	0.70	6.0x10^-3	100503890
Pemt	2.46	6.1x10^-3	18618
Snord99	0.52	6.1x10^-3	100217437
Snord67	0.39	6.2x10^-3	100217458
Snord12	0.60	6.2x10^-3	100217443
Nckap1l	2.23	6.3x10-3	105855
Adam8	0.64	6.4x10^-3	11501
Tmem140	14.72	6.4x10^-3	68487
Cntrob	0.78	6.4x10^-3	216846
Spaca6	0.77	6.4x10^-3	75202
Acr	0.55	6.4x10-3	11434
Araf	0.86	6.5x10^-3	11836
Gkap1	0.80	6.5x10^-3	56278
Myom3	0.04	6.6x10^-3	242702
Opn3	1.44	6.7x10^-3	13603
Gm17739	0.77	6.9x10^-3	319689
Gm19359	0.70	7.1x10^-3	100502768
Ascc2	0.79	7.1x10^-3	75452
Neurod6	1.52	7.2x10^-3	11922
Tspoap1	0.81	7.2x10^-3	207777
Srsf5	0.83	7.2x10^-3	20384
Gm15612	1.73	7.4x10^-3	100038356
Tmem28	1.54	7.4x10^-3	620592
Aifm3	0.34	7.4x10^-3	72168
Ttc19	0.85	7.5x10^-3	72795
Ctnnal1	0.78	7.6x10^-3	54366
Tmem17	0.73	7.6x10^-3	103765
Ankrd13d	0.74	7.6x10^-3	68423
NA	0.78	7.7x10^-3	100861853
Gbx1	2.20	7.7x10^-3	231044
Pygo2	1.16	7.8x10^-3	68911
Pnma1	1.86	7.8x10^-3	70481
Frem2	1.52	7.9x10^-3	242022

Brd9	0.86	7.9x10^-3	105246
Sema4d	1.29	8.0x10^-3	20354
Imp3	1.29	8.0x10^-3	102462
Vtn	1.66	8.0x10^-3	22370
Dnah1	0.75	8.1x10^-3	110084
Ints6l	0.80	8.1x10^-3	236790
Ahdc1	1.17	8.1x10^-3	230793
Gm19645	1.84	8.2x10^-3	100503344
Pla2g7	1.83	8.2x10^-3	27226
NA	1.83	8.2x10^-3	100861967
Zfc3h1	0.88	8.2x10^-3	216345
Snrnp70	0.88	8.3x10^-3	20637
Ubp1	0.85	8.3x10^-3	22221
Gm16440	3.04	8.3x10^-3	544998
NA	2.73	8.4x10^-3	100862026
Wsb1	0.79	8.4x10^-3	78889
Psmb7	0.87	8.5x10^-3	19177
Gm9385	0.77	8.6x10^-3	668829
Selenow	1.17	8.6x10^-3	20364
Lars2	0.86	8.6x10^-3	102436
Luc7l3	0.81	8.7x10^-3	67684
Atp6v0c	1.25	8.8x10^-3	11984
4930426I24Rik	28.48	8.8x10^-3	100504512
Gm20939	0.28	9.0x10^-3	100044193
NA	2.94	9.0x10^-3	100862121
Pcgf2	0.80	9.0x10^-3	22658
Alkbh5	1.15	9.0x10^-3	268420
Phc1	0.89	9.1x10^-3	13619
Atxn7l2	0.84	9.1x10^-3	72522
D330050I16Rik	2.08	9.1x10^-3	414115
Gm19592	1.49	9.2x10^-3	100503203
Hexdc	0.73	9.2x10^-3	238023
Snord34	0.48	9.3x10^-3	27210
Eml5	0.85	9.3x10^-3	319670
NA	3.85	9.3x10^-3	100502907
Cdk5r2	1.23	9.3x10^-3	12570

Ccnl2	0.81	9.4x10^-3	56036
Arhgap4	0.65	9.4x10^-3	171207
Pdxk	1.32	9.4x10^-3	216134
ND1	0.85	9.4x10^-3	17716
Pcgf5	1.85	9.6x10^-3	76073
Gpr173	1.22	9.7x10^-3	70771
Snord68	0.52	9.8x10^-3	100302565
F3	0.58	9.8x10^-3	14066
Gm19426	0.48	9.8x10^-3	100502874
Cep70	0.86	9.9x10^-3	68121
Pank4	0.77	9.9x10^-3	269614
Carmil3	0.82	9.9x10^-3	268747
Prpf4b	0.89	9.9x10^-3	19134
Ogt	0.85	9.9x10^-3	108155
